# Rif1 prolongs the embryonic S phase at the *Drosophila* mid-blastula transition

**DOI:** 10.1371/journal.pbio.2005687

**Published:** 2018-05-10

**Authors:** Charles A. Seller, Patrick H. O’Farrell

**Affiliations:** Department of Biochemistry and Biophysics, University of California San Francisco, San Francisco, California, United States of America; Dana-Farber Cancer Institute, United States of America

## Abstract

In preparation for dramatic morphogenetic events of gastrulation, rapid embryonic cell cycles slow at the mid-blastula transition (MBT). In *Drosophila melanogaster* embryos, down-regulation of cyclin-dependent kinase 1 (Cdk1) activity initiates this slowing by delaying replication of heterochromatic satellite sequences and extending S phase. We found that Cdk1 activity inhibited the chromatin association of Rap1 interacting factor 1 (Rif1), a candidate repressor of replication. Furthermore, Rif1 bound selectively to satellite sequences following Cdk1 down-regulation at the MBT. In the next S phase, Rif1 dissociated from different satellites in an orderly schedule that anticipated their replication. Rif1 lacking potential phosphorylation sites failed to dissociate and dominantly prevented completion of replication. Loss of Rif1 in mutant embryos shortened the post-MBT S phase and rescued embryonic cell cycles disrupted by depletion of the S phase–promoting kinase, cell division cycle 7 (Cdc7). Our work shows that Rif1 and S phase kinases compose a replication timer controlling first the developmental onset of late replication and then the precise schedule of replication within S phase. In addition, we describe how onset of late replication fits into the progressive maturation of heterochromatin during development.

## Author summary

Cells divide rapidly in the early embryos of most animals. However, during a conserved period of development known as the mid-blastula transition (MBT), the cell cycle slows down dramatically. In *Drosophila* embryos, genome duplication abruptly slows to initiate this cell cycle prolongation. This is achieved through the onset of late replication, a well-recognized phenomenon in which specific sequences of the genome await replication until long after other sequences have finished. Even though this temporal program of replication is a major determinant of the duration of S phase, the factors involved in this process remain unknown. Here, we use genetics and real-time microscopy to visualize replication in developing fly embryos and show that the protein Rap1 interacting factor 1 (Rif1) mediates the introduction of late replication at the MBT. We find that at this stage, Rif1 binds to and selectively delays the replication of large blocks of repetitive DNA known as satellite sequences. During the rapid cell cycles before the MBT, we show that the cyclin-dependent kinase 1 (Cdk1) prevents Rif1 from slowing down DNA replication by driving its removal from the chromatin. The developmental down-regulation of Cdk1 at the MBT allows Rif1 to associate with the satellite sequences and initiate cell cycle slowing. Our work provides new insights into the temporal programming of S phase and into the embryonic origin of late replication.

## Introduction

Eukaryotic DNA replication begins at many locations throughout the genome, known as origins. Different origins initiate at different times during S phase on a schedule governed by an elusive replication timing program. The time it takes to duplicate the genome, the length of S phase, is set by the time when the last sequence completes replication. For over 50 years, the field has appreciated that late replicating sequences are found in the compacted portion of the genome known as heterochromatin [1][2][3]. Late replication is presented as a general property of heterochromatin, but how this property arises is unknown [2]. Additionally, embryonic development of many animals features dramatic modifications of replication timing [4][5][6][7]. In *Drosophila*, the length of S phase changes by over 50-fold during development [8][9][10], and in the early *Drosophila* embryo, the heterochromatin does not replicate later than the rest of the genome. Late replication is then properly viewed as a feature that must be imparted to the heterochromatin at the beginning of every new generation. How S phase is retooled to do this is unknown.

As worked out in yeast, the process of origin initiation involves a sequence of conserved biochemical steps that converts an origin into a bidirectional replication fork [11][12]. Origins are first licensed for replication through the loading onto double-stranded DNA (dsDNA) of two helicase complexes composed of 6 minichromosome maintenance proteins (MCM2-7). The resulting head-to-head double hexamer is known as a pre-replicative complex (pre-RC). Activation of the pre-RC requires the coordinated assembly of a multiprotein complex called a replisome. Activation, also referred to as firing, is led by the action of 2 conserved cell cycle kinases: a cyclin-dependent kinase (CDK) and a Dbf4-dependent kinase (DDK) [13]. CDK and DDK are recruited to pre-RCs, where they phosphorylate substrates to initiate the transformation of the pre-RC into functional replication forks [14][15][16]. Local chromatin structure is thought to influence the efficiency of this step to alter the replication timing of different sequences [17]. Multiple studies have identified a number of pathways contributing to such local inputs, such as histone modification [18][19][20] and chromatin binding proteins [21][22]. Additionally, global factors, such as the competition among origins for limited replication components, have been suggested to impact timing [23][24]. However, we have little insight into how the varied inputs affecting the efficiency of pre-RC activation measure time to precisely schedule replication of different sequences during S phase or how this schedule is modulated during development.

In light of these issues, the embryo provides a unique context in which to study the control of S phase duration because development profoundly changes it. Historically, the study of cell cycle timing has focused on the G1/S and G2/M transitions, but the early embryonic cell cycles lack gap phases, and it is the duration of S phase that changes [25][26]. In the *D*. *melanogaster* embryo, these specialized cell cycles are maternally programmed, synchronous, and occur in the absence of cell membranes. During the first 9 nuclear cycles, S phase completes in as little as 3.4 min [8], and nuclei remain deep within the embryo. Beginning in cycle 10, the nuclei reach the surface of the embryo, forming the blastoderm. During cycles 11–13, the length of S phase gradually increases to 13 min. At the next cell cycle, the 14th, the embryo begins a conserved developmental period known as the mid-blastula transition (MBT) during which numerous changes occur: Interphase, which extends dramatically, has an S phase 14 that lasts 70 min, and it is followed by the first G2 [27][28]. After G2 of cycle 14, cells enter mitosis 14 in a patterned program controlled by zygotic transcription [29]. We focused on the changes that occur to the DNA replication program at the MBT and on how these changes are introduced. As we show here, the first introduction of late replication results from the emergence of a single regulatory input with timing function.

Prior work has shown that the prolongation of S phase at the MBT is caused by introduction of delays in the replication of satellite sequences [26]. During the preblastoderm cycles, all regions of the genome begin and end replication together, resulting in a short S phase. Even the 30% of the genome composed of blocks of repetitive DNA known as the satellite sequences, which are considered to be constitutively heterochromatic, lacks the marks of heterochromatin and replicates early. During S phases 11–13, the satellite sequences experience progressively modest delays in replication timing. Then, these satellites experience major delays in S phase 14 in which a succession of different satellites begin and complete replication on a protracted schedule. Thus, onset of late replication causes the pronounced increase in the S phase duration, but the nature of the timer coordinating this schedule was unknown. Following their late replication in cycle 14, the satellite sequences become heterochromatic, and late replication will be a characteristic feature of heterochromatic satellites for the rest of development.

Uncovering how late replication develops will improve our understanding of what has been a widespread but mysterious feature of DNA replication. We know that the onset of late replication in the embryo follows a time course that is unrelated to appearance of other features of heterochromatin such as compaction, which is already evident in earlier cell cycles, or heterochromatin protein 1 (HP1) recruitment, which occurs after replication in cycle 14, or histone H3 methylated on lysine 9 (H3K9me), which accumulates continuously and slowly over the course of interphase 14 [26][30]. Previous work demonstrated that activity of the cyclin-dependent kinase 1 (Cdk1) kinase is a key determinant of the duration of S phase in the early embryo [31]. Persistent S phase Cdk1 activity during the shorter pre-MBT cycles drives heterochromatin to replicate early, and it is the programmed down-regulation of Cdk1 occurring at the MBT that allows the onset of late replication. However, it is unclear how the embryo interprets the activity of Cdk1 to produce this dramatic prolongation of S phase.

We have discovered that Rap1 interacting factor 1 (Rif1)—a conserved protein that interacts with protein phosphatase 1 (PP1) and impacts telomere biology, DNA damage responses, and replication timing [32][33][34][35]—is an important regulator of early developmental changes in cell cycle timing. Amid reports of diverse roles for Rif1, several studies emphasize the action of Rif1 to inhibit replication and show that Rif1 and DDK oppose each other, inhibiting or activating the pre-RC, respectively [36][37][38][39]. The literature has emphasized the possibility that Rif1-recruited PP1 could counter the action of DDK by dephosphorylating this kinase’s substrate, the MCM helicase complex, but genetic epistasis and molecular findings suggest that DDK also targets and inactivates Rif1, suggesting that a more complex interplay of the activating kinases and the Rif1 inhibitor contributes to pre-RC activation [32]. While it is appreciated that Rif1 and its associated PP1 can inhibit the firing of origins, the eventual firing of these inhibited origins presumably requires an input to negate or override the repressive action of Rif1. The nature of this signal, which would underlie the temporal programming for the late-replicating origins, is unknown. Additionally, how Rif1 contributes to developmental changes in replication timing is unknown. Our results describe how Rif1 connects the activity of Cdk1 to the onset of late replication in the embryo, thereby composing an active program that extends S phase at the *Drosophila* MBT. We provide new insights into Rif1 behavior that suggest an intimate role in a timing mechanism specifying the precise time at which different sequences replicate during S phase. In addition, our work allows us to provide a more complete description of the maturation of the heterochromatin during development.

## Results

### Rif1 binds to compacted foci of satellite sequences as the length of the embryonic S phase increases

**Fig 1:**
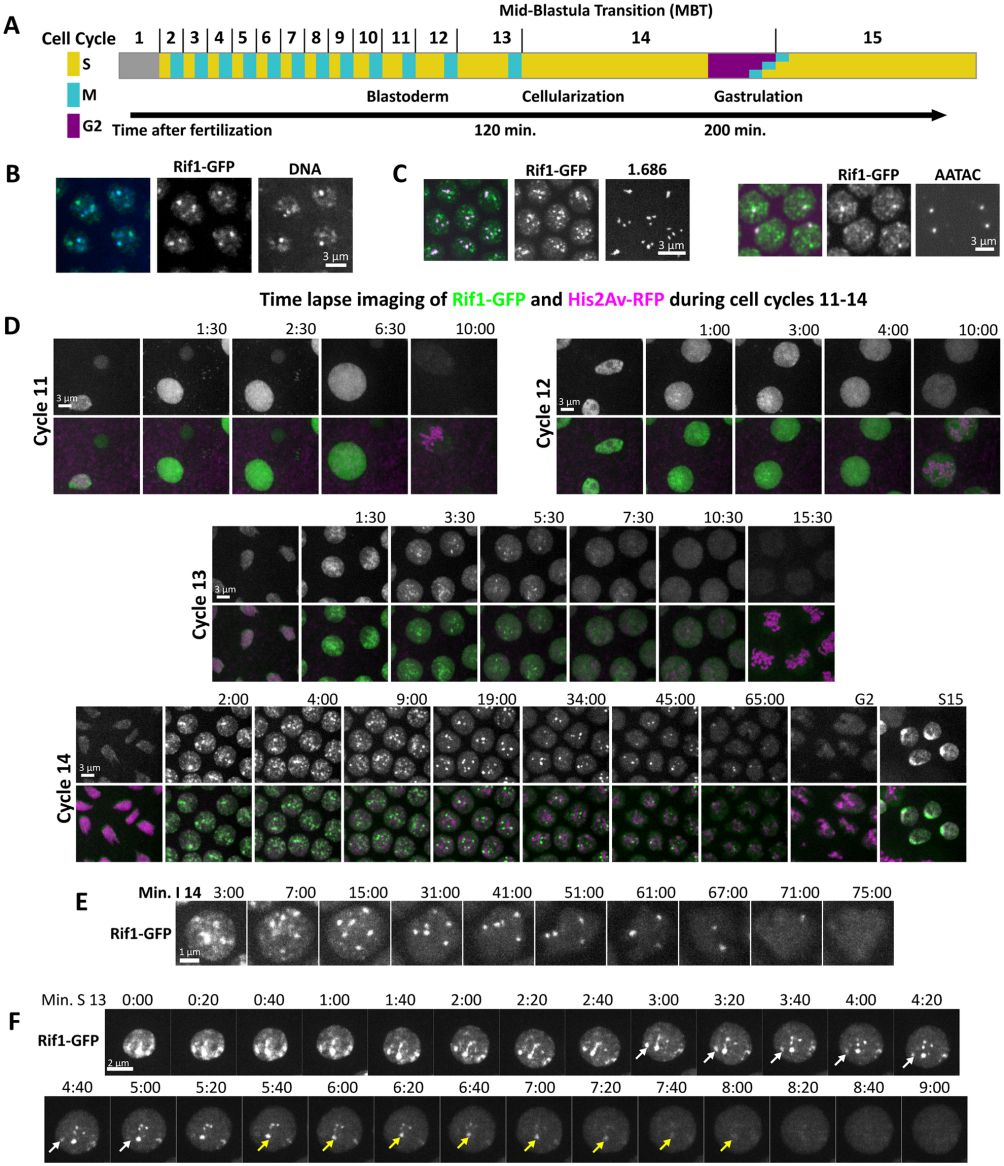
Rif1 forms foci on heterochromatin as the embryonic S phase lengthens. (A) Diagram highlighting the key changes to the cell cycle and to embryonic morphology during early development. The initial prolongation of the cell cycle is due to the increase in the length of S phase. This occurs gradually during cycles 11–13, and then the onset of a late-replication program in S phase 14 extends interphase considerably. The first G2 is introduced in cycle 14, after which cells enter mitosis 14 asynchronously, according to a developmental pattern. (B) Nuclei from an S phase 14 Rif1-GFP embryo stained for GFP (green in the merge) and DNA (DAPI: blue in the merge). Rif1 colocalizes with regions of the interphase nucleus that stain intensely with DAPI. (C) Specific satellites (magenta in the merge), either 1.686 detected live by TALE-light or AATAC detected by DNA FISH colocalized with Rif1-GFP (green in merge) during early cycle 14. (D) Frames from live imaging (accompanied by S1 Movie) showing Rif1-GFP and His2Av-RFP in developing embryos during cell cycles 11–14. Note the initial presence and increasing persistence of nuclear Rif1 foci as interphase lengthens. (E) Magnified images from time-lapse records of Rif1-GFP during S phase 14. Individual nuclear foci of Rif1 disappeared at different times across S phase. (F) Frames from time lapse showing Rif1-GFP in a single nucleus at 20-s intervals during S phase 13 (accompanied by S2 Movie). Arrows indicate 2 specific foci at the time that their fluorescence declined. FISH, fluorescence in situ hybridization; GFP, green fluorescent protein; MBT, mid-blastula transition; His2Av, histone 2A variant; RFP, red fluorescent protein; Rif1, Rap1 interacting factor 1; TALE, transcription activator-like effector.

Because of the stereotyped nature of the embryonic cell cycles (summarized in Fig 1A), live imaging of fluorescently tagged proteins can be especially informative. We used clustered regularly interspaced short palindromic repeat (CRISPR) genome editing to tag the endogenous Rif1 protein with enhanced green fluorescent protein (EGFP) at its C-terminus (S1 Fig). Rif1 is maternally provided (S1 Fig) and is widely distributed during the first 6 h of embryogenesis and thereafter shows increasingly tissue-limited expression [40].

Given ubiquitous presence of Rif1 protein in the early embryo, regulation of its activity ought to underlie any developmental or cell cycle modulations of Rif1 function at this stage. If Rif1 acts to delay the replication of heterochromatin, then we would expect the protein to be recruited to satellite sequences when the replication of these sequences is delayed in cycle 14. The satellite sequences form compacted regions of chromatin that can be visualized as discrete bright foci of DAPI-stained DNA (Fig 1B). These compacted foci of chromatin first acquire heterochromatic marks during cycle 14, but given the absence of G1 in this cycle, a program for their delayed initiation must already be in place at the beginning of interphase 14 [26][30]. In S phase 14, Rif1 was bound to many foci of compacted chromatin, while in the following G2 phase, the heterochromatin lacked Rif1 staining (Fig 1). Live imaging of Rif1-EGFP embryos (S1 Movie) during early embryogenesis revealed the dynamics of this change. Rif1-EGFP disappeared from individual foci as S phase progressed, and only a dim nuclear background was present by G2 of cycle 14 (Cycle 14, Fig 1D). Tracking individual foci in high-frame-rate movies showed that different Rif1 foci disappeared at different times (Fig 1E). Often, only a single focus remained near the end of S phase before disappearing as cells entered G2. This ordered loss of different Rif1 foci was reminiscent of the protracted schedule of late replication occurring in this cycle.

To test the correspondence of Rif1 foci and satellite sequences, we marked specific satellite sequences by in situ hybridization or our recently developed transcription activator-like effector (TALE)-lights technique [41]. In embryos fixed during early S phase 14, the single Y-chromosome-linked focus of AATAC satellite detected by DNA–fluorescence in situ hybridization (FISH) was costained by Rif1 (right panels Fig 1C). In live embryos, a fluorescently tagged TALE-light protein engineered to bind the late replicating satellite-repeat 1.686 showed that Rif1 also binds the 4 foci of this sequence (left panels in Fig 1C).

During cycles 11–13, S phase gradually and progressively gets longer due to incremental delays to satellite replication. In parallel with these changes, Rif1 showed weak and transient localization to foci in cycle 11 and progressively more intense and longer-lived foci in subsequent cycles (S1 Movie, Fig 1D). Disappearance of Rif1 foci within each S phase was not instantaneous: The signal decayed from the outside-in over a few frames of our records; for instance, during S phase 13, high-frame imaging showed that a focus of Rif1 disappeared over the course of approximately 2 min (S2 Movie, Fig 1F). These observations show that Rif1 binds to satellite sequences as they become late replicating during embryogenesis and that Rif1 dissociates from satellite sequences as replication progresses.

### The release of Rif1 from chromatin anticipates the initiation of late replication

**Fig 2:**
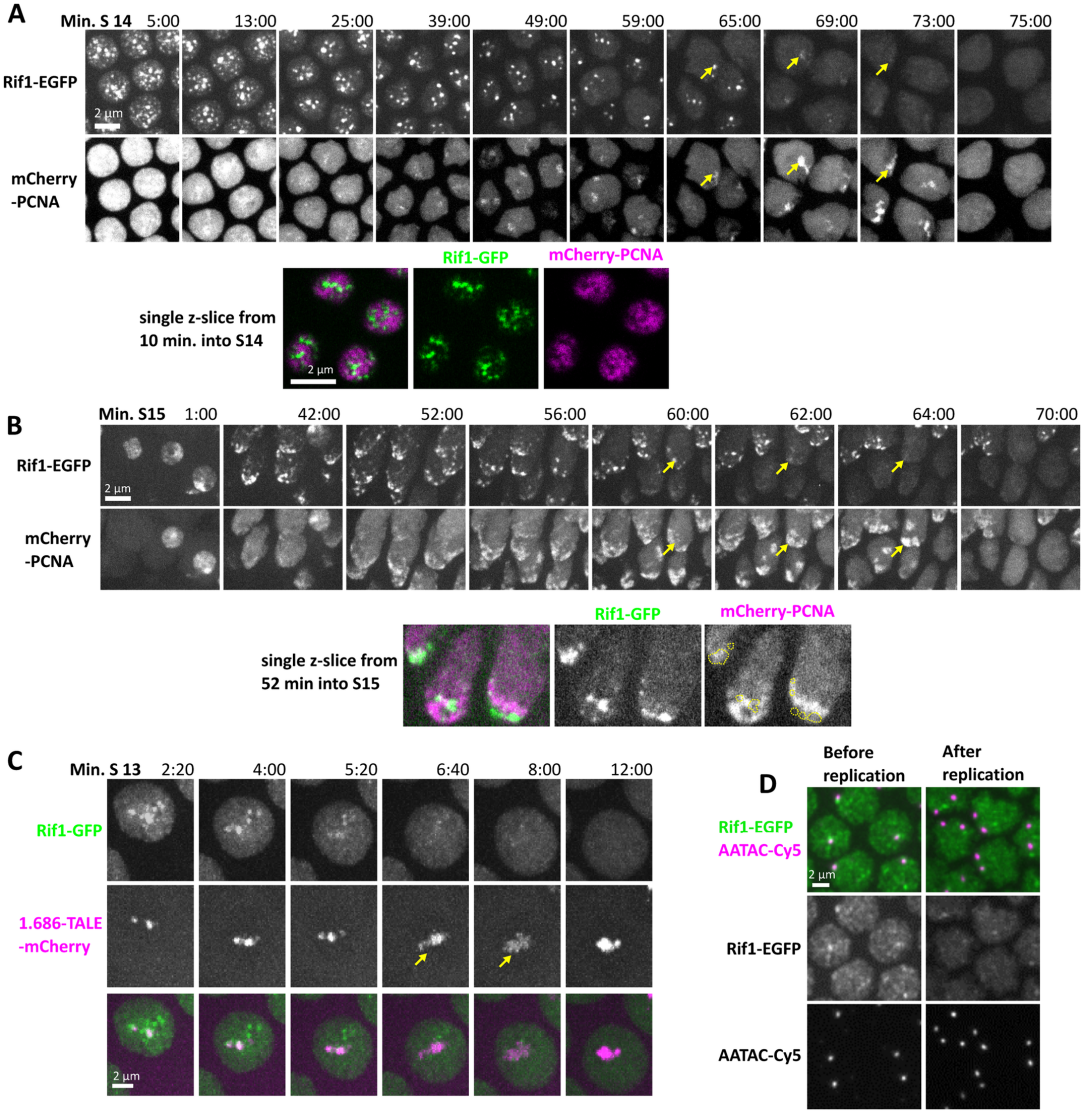
Rif1 dissociates from chromatin before the underlying sequences replicate. (A) Stills from time-lapse microscopy of Rif1-GFP and mCherry-PCNA during S phase 14 (S3 Movie). Rif1 marks numerous satellites early, and the number of foci progressively declines. After early widespread staining, PCNA, a marker of active replication, transiently marks a progression of different satellites. Though both Rif1-GFP and mCherry-PCNA form foci on satellite sequences, the signals do not overlap. At different positions, the period of PCNA recruitment follows loss of Rif1. For example, the yellow arrows track a Rif1 focus as it disappears. In the paired mCherry-PCNA images, recruitment occurs just as Rif1 disappears. Note that the mCherry-PCNA signal is much expanded, in agreement with previous findings that PCNA recruitment is coincident with decompaction of satellite foci [26]. Below, a single z plane from 10 min into S phase 14 shows that even at this early stage of S phase when mCherry-PCNA is widespread, there are “holes” in its distribution, and the signal from Rif1 falls within these holes. (B) Stills from time-lapse microscopy of Rif1-GFP and mCherry-PCNA during S phase 15 (S5 Movie). During this S phase, the late-replicating heterochromatin is clustered into an obvious chromocenter. Within this cluster, replicating and nonreplicating satellites are nearby, but each satellite forms a coherent focus and replicates at different times [26]. As in S phase 14, Rif1 marks late-replicating chromatin, and Rif1 dissociates from these sequences before the acquisition of PCNA. Yellow arrows track the dissociation of Rif1 from one focus, followed by its recruitment of mCherry-PCNA as it decompacts and replicates. Below, a single z plane from 52 min into S phase 15 shows that Rif1 does not colocalize with PCNA. The outline of Rif1 foci is traced in yellow on the still image of PCNA. Note that the single-plane images do not show all of the nuclei seen in the merged stack shown above in (B). (C) Live imaging of Rif1 and the satellite 1.686 using an mCherry-tagged TALE protein (S6 Movie). Yellow arrow indicates the decompaction and replication of 1.686. After its replication, the 1.686 repeat recompacts and appears brighter. Rif1 dissociates from this sequence prior to its replication. (D) Nuclei in S phase 14 stained for Rif1 and for the Y-chromosomal satellite repeat AATAC. Rif1 is bound to AATAC during early S14 before the AATAC repeat replicates. During late S14, the duplicated AATAC sequence lacks Rif1 stain. EGFP, enhanced green fluorescent protein; GFP, green fluorescent protein; PCNA, proliferating cell nuclear antigen; Rif1, Rap1 interacting factor 1; TALE, transcription activator-like effector.

The correlation between the dissociation of the Rif1 localized to satellite foci and the onset of late replication motivated a closer examination of Rif1 dynamics during S phase. We have previously validated and utilized fluorescently tagged versions of the replication protein proliferating cell nuclear antigen (PCNA) as real-time probes for the progress of S phase [25]. PCNA travels with the replicating DNA polymerase, and its recruitment to different regions of the genome marks their replication. Using a transgenic line expressing an mCherry-labeled version of PCNA from its endogenous promoter (S2 Fig), we were able to compare active DNA replication with the localization of Rif1. During the first 10 min of S phase, the PCNA signal was intense and distributed throughout most of the nucleus. Following early replication, the generalized signal fades progressively, and the PCNA signal is predominantly limited to bright apical foci, which mark the late-replicating satellite sequences. Each late-replicating focus appears, persists, and declines in a stereotyped schedule, with the number of active foci gradually declining during more than an hour of interphase of cycle 14 (Fig 2A, S3 Movie). Throughout this program, Rif1 did not overlap with the PCNA signal, indicating that once a region had initiated DNA replication, the Rif1 signal had already declined to background levels. As S phase progressed, different Rif1 foci disappeared at different times and were replaced within 1 min by PCNA (S3 Movie). The last sequence to recruit PCNA was marked by Rif1 throughout S phase 14 until just before it recruited PCNA (Fig 2A). Similar analyses in cycles 12 and 13 similarly revealed separation of Rif1 staining and replication. The observed timing suggested that replication of Rif1 staining regions awaits the dissociation of Rif1. Additionally, a prior study of fixed mammalian cells documented that Rif1 does not colocalize with DNA stained by incorporation of labeled nucleotides, indicating that this dynamic nature of Rif1 localization is conserved [33].

The S phase of cell cycle 15 provides an alternative context to observe the relationship between Rif1 and replication. By cycle 15, the embryo has completed the MBT, the cell cycle has lost synchrony, and morphogenetic movements have begun. The timing of mitosis 14 and entry into cycle 15 differs with position in the embryo but follows a stereotyped schedule that is zygotically controlled. Cycle 15 still lacks a G1, so nuclei enter S phase immediately following mitosis 14, but these cycle 15 nuclei exhibit more mature features. The satellite sequences enter cycle 15 after introduction of heterochromatic marks such as H3K9me and localized HP1 during cycle 14 [26]. Furthermore, the distinct foci of satellite sequences seen at the beginning of cycle 14 are merged in an easily identifiable chromocenter in the apical part of the nucleus. Despite these differences, a connection between Rif1 and the replication program was still observed. At late anaphase of mitosis 14 and the onset of cycle 15, Rif1 was recruited rapidly to separating chromosomes and appeared especially bright over the leading edge of the advancing chromosomes, the site of pericentric satellite sequences that constitute the bulk of the heterochromatin. As the telophase nucleus formed, bright Rif1 signal was seen over the compacted chromatin of the chromocenter before the recruitment of PCNA to the nucleus (S4 Movie, S2 Fig). At the onset of S phase 15, PCNA was recruited only to euchromatic regions, and it did not overlap Rif1 (Fig 2B). As S phase 15 progressed, PCNA was recruited to the edge of the compacted heterochromatin, where we have previously observed decompaction of HP1-staining chromatin [26]. Although aggregated in a single mass in the nucleus (the chromocenter), individual satellite sequences remain as distinct subdomains and retain an individual replication schedule [26]. In agreement with this, Rif1 staining was progressively limited to more restricted regions within the chromocenter, with latest Rif1 foci dispersing toward the end of S phase (Fig 2B, S5 Movie). Real-time records again documented a close connection between the loss of Rif1 from chromatin and the recruitment of PCNA to the underlying region.

Having followed the progress of S phase globally, we next wanted to examine the replication of a specific heterochromatic sequence, the 1.686 satellite repeat. The simple repeated sequence 1.686 is found at 4 loci, one to the left and one to right of the centromeres of both chromosomes 2 and 3. All these foci replicate together and show characteristic delays in their time in cycles 13 and 14. TALE-light probes can be injected into embryos and used to track the repetitive DNA in real time [30] [41]. Purified mCherry-labeled TALE-light protein recognizing the 1.686 repeat was injected into syncytial embryos and filmed. As reported previously, the TALE-light was gradually recruited to 1.686 during interphase, with the signal appearing as compact foci. During replication, the TALE-light signal became noticeably fuzzier, presumably due to the decompaction of the heterochromatic DNA during its active replication [41]. Upon completion of replication, the TALE-light signal was again compact and obviously brighter. We use this reproducible behavior to indirectly follow the replication of 1.686. During cycle 13, we observed that Rif1 was recruited to 1.686 at the beginning of S phase and disappeared immediately before the decompaction and replication of the repeat. We observed intermediate intensities of Rif1 signal on 1.686 in the minute preceding its decompaction. Rif1 dissociation was relatively rapid but progressive, with the Rif1 signal decaying over the minute preceding 1.686 decompaction. After completing replication, 1.686 lacked Rif1 for the remainder of the cycle (Fig 2C, S6 Movie). Thus, Rif1 dissociates from this specific satellite immediately prior to the onset of its replication. We also examined fixed embryos using FISH probes to localize another heterochromatic repeat, AATAC, and showed that Rif1 was bound to this repeat AATAC during early S phase 14, but no such signal was observed in embryos aged to later in S phase after the replication of this sequence (Fig 2D). These observations show that Rif1 dissociates from specific satellite sequences upon their replication.

Our observations reveal that the dynamics of Rif1 interaction with chromatin parallel changes in replication timing. Rif1 association to satellite sequences began when these sequences first showed a slight delay in replication. While association was transient in the earlier cycles, Rif1 associated more persistently with satellites during the much-extended S phase of cycle 14. Most dramatically, Rif1 dissociated from individual satellite sequences at distinct times that align with the onset of PCNA recruitment to those sequences. The disappearance of the last foci of Rif1 staining marked the onset of replication of the latest replicating sequence and anticipates the end of S phase. These parallels suggest that binding of Rif1 to sequences might defer their replication until its dissociation. If so, analysis of this interaction may give us insights into the regulation of late replication and S phase prolongation.

### Steps in the appearance of localized Rif1 in foci

Late-replicating sequences have to be specified before the onset of replication if they are to avoid early firing. In yeast and mammalian cells in culture, this is thought to occur well before replication at a critical time during G1, known as the timing decision point [42][43]. However, in the G1-less embryonic cell cycles, the preparation for S phase is compressed and overlaps mitosis. Since cyclin:Cdk1 activity inhibits preparation of origins for replication, all of the preparations for replication happen between the down-regulation of cyclin:Cdk1 at the metaphase–anaphase transition and the onset of S phase upon entry into the next interphase. Real-time observation of green fluorescent protein (GFP)-tagged origin recognition complex 2 (Orc2) protein revealed that Origin Recognition Complex (ORC) binds to the separating anaphase chromosomes prior to the midpoint of their separation [44]. The MCM helicase is loaded shortly later in anaphase [45]. Replication begins at mitotic exit without obvious delay. This suggests that at the time of the transition from mitosis to interphase, there is already some type of feature that distinguishes the satellites so that they do not begin replication immediately [26]. If Rif1 is to delay the replication time of specific sequences, we expect its recruitment to chromatin to occur during this period. Indeed, we observed binding of Rif1 to separating anaphase chromosomes before the recruitment of PCNA to the nucleus, which marks the start of interphase (S2 Fig). However, as described below in cycles prior to cycle 15, the specificity of Rif1 binding to satellite sequences emerges in concert with progression into interphase.

**Fig 3:**
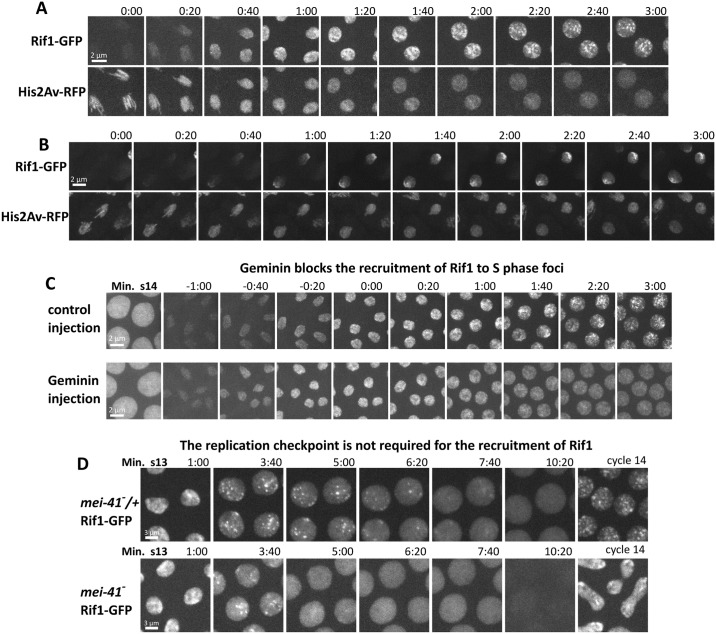
Two stages of Rif1 recruitment, their contributions to specificity, and reliance on origin licensing and the replication checkpoint. Time-lapse confocal microscopy on Rif1-GFP, His2Av-RFP embryos showing the initial binding of Rif1 during transit from one cell cycle to the next. (A) During late anaphase 13, on the approach to cycle 14, chromosomes first exhibit faint and ubiquitous Rif1 staining that accumulates for about 1 min. During the following minute as S phase begins, accumulation continues but is now localized to foci that become clearer as the nuclei swell. See also S2 Movie. (B) During late anaphase 14, on the approach to cycle 15, the early stage of Rif1 staining shows some specificity for the pericentric regions of the chromosomes and forming chromocenter. This specificity is amplified as the interphase 15 nucleus forms and S phase begins. (C) Time-lapse imaging of Rif1-GFP during the normal transition from cycle 13 to 14 and in an embryo injected with purified geminin protein in interphase of 13. Times are indicated with reference to the start of S phase 14. The geminin block to pre-RC formation prevented the recruitment of Rif1 to foci but did not block the initial generalized binding during mitotic exit. (D) Imaging of Rif1-GFP during cell cycle 13 in control (*mei41/+*) and *mei41*-null embryos. The Mei41-dependent replication checkpoint is essential during cycle 13 to prevent premature entry into mitosis (10:20 frame). Rif1 foci still form in the absence of a replication checkpoint, but the Rif1 foci are lost earlier, and the premature mitosis leads to bridging and defective cycle 14 nuclei. GFP, green fluorescent protein; His2Av, histone 2A variant; pre-RC, pre-replicative complex; RFP, red fluorescent protein; Rif1, Rap1 interacting factor 1.

We examined the initial binding of Rif1 by filming mitosis 13 and early interphase 14. We observed an abrupt onset of faint and generalized binding of Rif1 to the chromosomes during late anaphase/telophase as previously observed in fixed samples (Fig 3A) [34][40][46]. Rif1 accumulation continued as the telophase nucleus formed. Although little was resolved in the compact and brightly staining telophase nucleus, by 1 min into S phase, brighter foci of staining became clear, and much of the nuclear signal quickly declined except in foci clustered at the apex of the nucleus where the foci of pericentric satellite sequences lie (S2 Movie, Fig 3A). Thus, while a low level of generalized binding is clear in mitosis, specificity in the binding became evident only in the beginning of interphase, suggesting that there are two phases of binding. In contrast, in the transition to cycle 15, the initial binding of Rif1 in anaphase, while still weak, was not uniform. During late anaphase 14, Rif1 was clearly enriched at the leading region of the chromosome mass, where the pericentric heterochromatin resides (Fig 3B, S4 Movie). This localization to the heterochromatic region was visualized without interruption as Rif1 continued to accumulate with entry into interphase 15, which is accompanied by immediate onset of replication as this cycle still lacks a G1 phase. Thus, the character of the initial anaphase binding of Rif1 changes between anaphase 13 and anaphase 14. Satellite sequences acquire some of the markings of heterochromatin, such as methylation and HP1 localization, during interphase of cycle 14. These heterochromatic marks might then guide the initial interaction of Rif1 at anaphase 14, but they would not be available to do so in anaphase 13.

Because recruitment of Rif1 began late in anaphase, just after the time when Orc2 was recruited to chromosomes, we wondered if pre-RC formation might be involved in Rif1 binding. Indeed, prior work in a *Xenopus* extract system suggests that this is true [47]. To test this, we examined Rif1 recruitment after the injection of embryos with purified geminin protein during mitosis 13. Geminin blocks the formation of the pre-RC by inhibiting Cdt1, a key helicase-loading factor, and prevents subsequent replication. Injection of geminin did not block the initial generalized binding of Rif1 to the late anaphase chromosomes or its nuclear accumulation in telophase nuclei but did block the emergence of localized Rif1 foci. Following geminin treatment, Rif1 was diffusely localized throughout the nucleus during interphase 14. (Fig 3C). We conclude that geminin blocks the localization of Rif1 to late-replicating sequences but emphasize that the results do not necessarily imply a direct involvement of pre-RC formation in this localization. Because geminin did not affect initial binding of Rif1, it might be the downstream consequences of a failure to assemble pre-RCs that produces the observed result.

### Recruitment of Rif1 to satellite sequences prior to the MBT does not require the replication checkpoint activity

Beginning in cycle 11, the gradual lengthening of interphase depends in part on the conserved DNA replication checkpoint [26][48][49]. During cycle 13, the checkpoint is required to delay activation of Cdk1 during S phase and prevent premature entry into mitosis. Embryos mutant for the checkpoint kinase ataxia telangiectasia and Rad3-related protein (ATR; *mei41*) fail to delay mitosis 13 sufficiently and so enter a catastrophic mitosis before the completion of replication, resulting in massive chromosome bridging. Because Rif1 foci first became evident during these gradually slowing cycles, we wondered if the replication checkpoint might impact Rif1 recruitment. However, in *mei41* embryos, both the initial binding of Rif1 during anaphase of M12 and its subsequent recruitment to nuclear foci in interphase 13 were indistinguishable from control embryos (Fig 3D). We did observe that the disappearance of Rif1 foci was accelerated in *mei41* embryos, and Rif1 was lost from chromatin before entry into a catastrophic mitosis 13. We conclude that the recruitment of Rif1 to late-replicating sequences is independent of the replication checkpoint but that timing of Rif1 dispersal is accelerated in its absence.

### Cdk1 activity promotes Rif1 release from chromatin during S phase

Prior work demonstrated that down-regulation of Cdk1 activity at the MBT plays a key role in extending S phase [30][31][50]. Cdk1 activity during the earlier rapid cell cycles was shown to be required for satellite sequences to replicate early and to sustain short S phases. The gradual decline in mitotic activators during cycles 11–13 contributes to the progressive lengthening of S phase. Finally at the MBT, the abrupt drop in Cdk1 activity is required for late replication and the resulting prolongation of S phase. These changes to Cdk1 parallel the changes we observed for Rif1 localization, so we investigated the connection between them.

**Fig 4:**
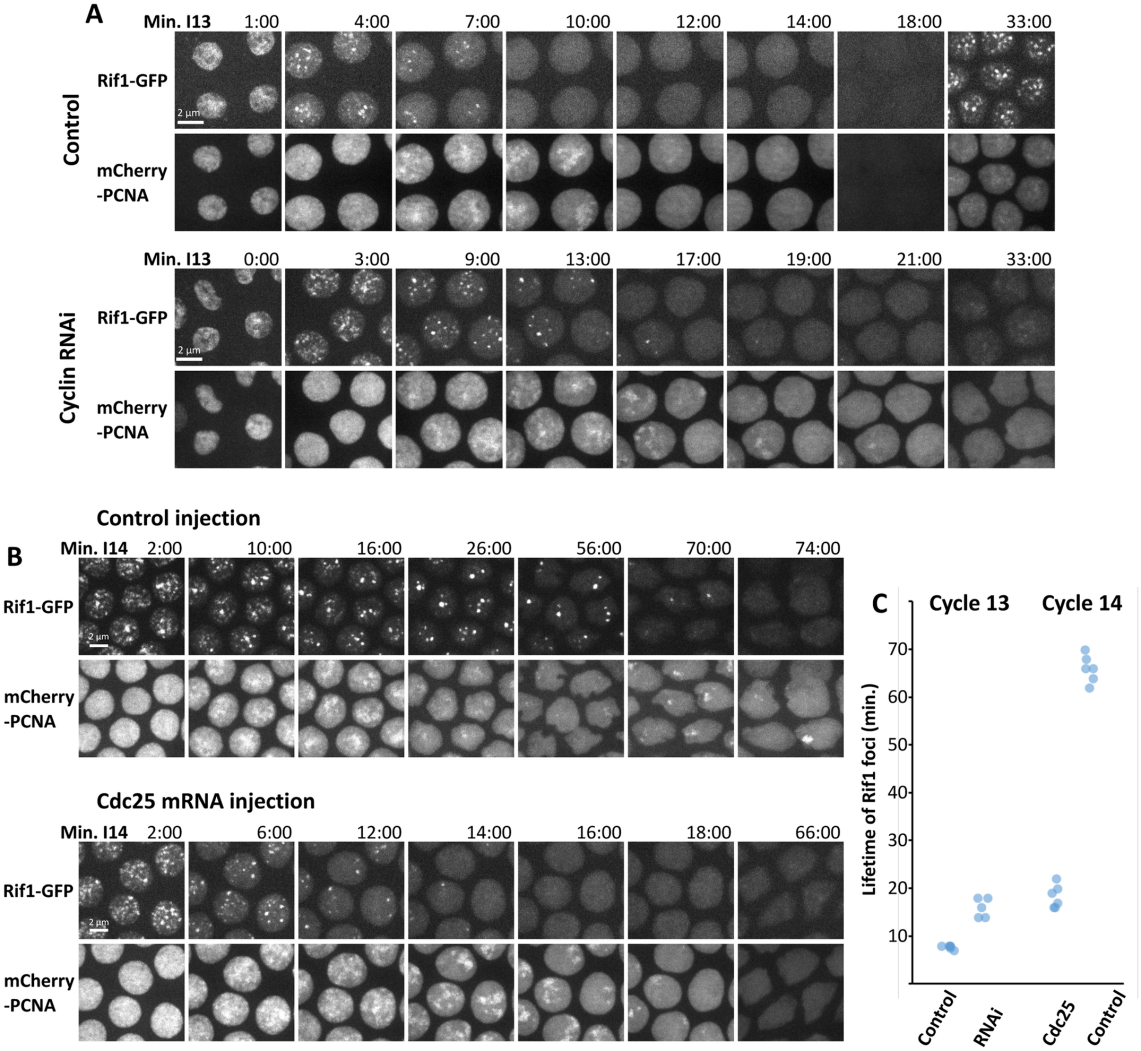
Manipulation of Cdk1 activity alters the lifetime of Rif1 foci and the duration of S phase. (A) Stills from time-lapse imaging of Rif1-GFP and mCherry-PCNA during cycle 13 after injection of either buffer (control) or dsRNA against the three mitotic cyclins (A, B, and B3) in cycle 10. The knockdown of the cyclins increases the persistence of Rif1 foci, extends S phase (PCNA staining), and blocks progression to cycle 14. The time of the last frame showing a Rif1 focus is indicated as the lifetime of Rif1 foci in (C). (B) Stills from time-lapse imaging of Rif1-GFP and mCherry-PCNA during cycle 14 after injection of either buffer (control) or mRNA encoding the Cdk1 activator Cdc25 (twine) in cycle 13. Artificial activation of Cdk1 during S phase 14 accelerates both the dissociation of Rif1 and late replication. In the control embryo, Rif1 foci were detected 70 min into S phase 14, and prominent foci of PCNA were documented at 74 min. Expression of Cdc25 resulted in the loss of Rif1 foci by 16:00, and the final late-replicating PCNA signal documented was at 18:00 (S7 Movie). (C) Plot of lifetime of Rif1 foci in minutes during either S phase 13 or S phase 14 following the indicated injection (S1 Data). Cdc25, cell division cycle 25; Cdk1, cyclin-dependent kinase 1; dsRNA, double-stranded RNA; GFP, green fluorescent protein; PCNA, proliferating cell nuclear antigen; Rif1, Rap1 interacting factor 1; RNAi, RNA interference.

Experimental reduction of Cdk1 activity by injection of dsRNA to induce RNA interference (RNAi) against the 3 mitotic cyclins (A, B, and B3) during cycle 10 arrests embryos in interphase 13 and extends the length of S phase 13 from 13 min to an average of 19 min [31]. Cyclin knockdown also affected the dynamics of Rif1 chromatin association. In arrested embryos, foci of Rif1 binding persisted for an average of 16 min, compared to 8 min in control-injected embryos (Fig 4A and 4C). This delay in the loss of Rif1 foci paralleled, and slightly preceded, the delayed appearance of late-replicating PCNA signal. In contrast, as previously reported, increasing Cdk1 activity during cycle 14 by injecting mRNA for the mitotic activator cell division cycle 25 (Cdc25) shortened S phase from over 1 h to an average of 22 min and can result in an early mitosis 14, with no bridging, indicating that DNA replication was complete. Experimental Cdk1 activation also changed the Rif1 program. In Cdc25-injected embryos, foci of Rif1 association were evident for only an average of 18 min compared to 66 min in control-injected embryos (Fig 4B and 4C). Real-time records documented that ectopic Cdk1 activity removed Rif1 from chromatin, allowing the underlying satellite DNA to initiate replication without the characteristic delays normally observed in S phase 14 (S7 Movie, Fig 4B). We conclude that Cdk1 activity can accelerate both the release of Rif1 from chromatin and the late-replication program. Both experiments demonstrate that the timing of Rif1 dissociation from chromatin and the subsequent initiation of late replication are sensitive to the activity of Cdk1 during S phase.

These results show that Cdk1 activity in early cycles normally promotes Rif1 dissociation from chromatin in pre-MBT embryos and that an artificial increase in Cdk1 in cycle 14 can do the same. The parallel effects of the experimental manipulations on Rif1 association and the progress of S phase further support suggestions that Rif1 association suppresses replication and that activation of origins in satellite sequences occurs in conjunction with Rif1 dissociation. The influence of Cdk1 on Rif1 suggests that the developmental program of Cdk1 down-regulation guides the observed changes in Rif1 dynamics and thereby S phase duration. In light of these findings, we can interpret the accelerated loss of Rif1 in *mei41* embryos (Fig 3D) as a consequence of the faster activation of S phase Cdk1 in the absence of the replication checkpoint [51]. These observations suggest that activity of the Cdk1 kinase directly or indirectly regulates Rif1 interaction with chromatin.

### A phospho-site mutant Rif1 does not release from heterochromatin and prevents completion of DNA replication

**Fig 5:**
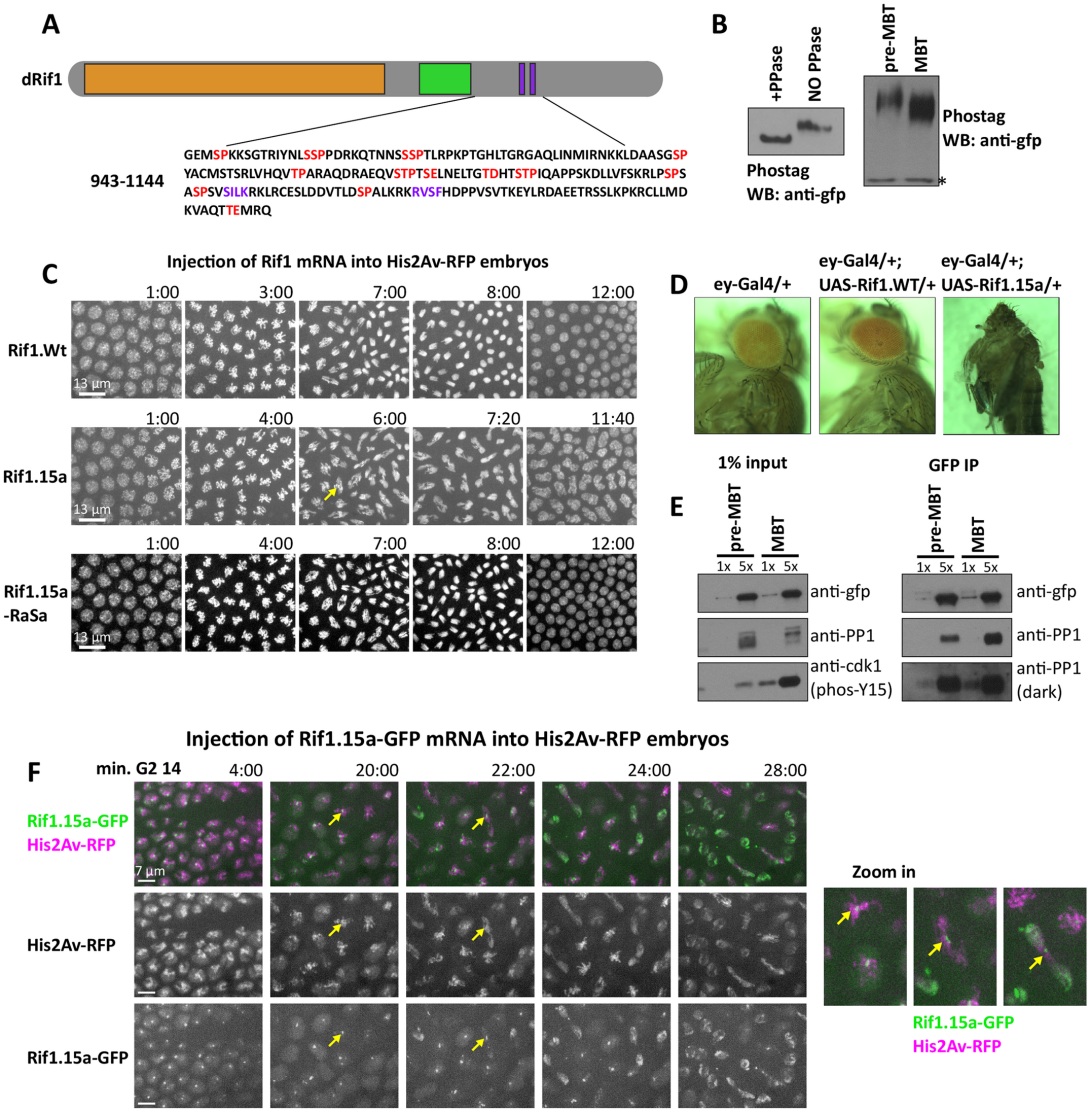
Phospho-site mutant Rif1 does not dissociate from chromatin and blocks replication. (A) Schematic (above) of Rif1 protein with N-terminal HEAT repeats (orange), putative DNA-binding domain (green), and PP1 interaction motifs (purple boxes). The amino acid sequence (below) of the indicated portion of the Rif1 protein shows candidate CDK and DDK phosphorylation sites (red). These 15 S/T residues were mutated to alanine to create the Rif1.15a phospho-mutant allele. (B) Left panel shows anti-gfp WBs used to detect Rif1-GFP from 1-h-old embryos. Protein extracts were treated with lambda phosphatase or buffer only for 1 h and then were run on SDS-PAGE gels cast with phos-tag to decrease the migration of phosphorylated proteins. Right panel shows phos-tag WBs used to detect Rif1-GFP from embryos aged to 1 h (pre-MBT) or to 2.5 h (MBT). Asterisk denotes background band. (C) Ectopic expression of the indicated versions of Rif1 by injection of mRNA during cycle 11. After injection, embryos were filmed during mitosis 13. The yellow arrow points to an example of an anaphase bridge. (D) Images of flies (left) and a pharate pupa (right) show the consequence of expression of either Rif1 (middle) or Rif1.15a (right) in the developing fly eye/head capsule using Eyeless-Gal4. (E) Anti-GFP immunoprecipitation followed by western blotting with the indicated antibodies. Protein extracts were prepared from embryos harvested before the MBT (1 h old) or after aging to the MBT (2.5 h old). Phos-Y15 (left-bottom) indicates the inactive phosphorylated form of Cdk1. (F) Frames from live imaging of nuclei starting in G2 of 14 and progressing into a defective mitosis following expression of Rif1.15a-GFP from injected mRNA. Yellow arrows indicate extensive anaphase bridging with a residue of Rif1.15a-GFP. Magnified images (right) show progression of one nucleus. CDK, cyclin-dependent kinase; DDK, Dbf4-dependent kinase; GFP, green fluorescent protein; His2Av, histone 2A variant; IP, immunoprecipitation; MBT, mid-blastula transition; PP1, protein phosphate 1; RFP, red fluorescent protein; Rif1, Rap1 interacting factor 1; UAS, upstream activating sequence; WB, western blot; WT, wild type.

Work from both *Saccharomyces cerevisiae* and *Schizosaccharomyces pombe* indicates that the kinases CDK and DDK act on conserved phosphorylation sites to inhibit Rif1 and limit its ability to inhibit replication [37][38]. In these yeasts, phosphorylation of Rif1 is thought to block Rif1’s ability to recruit PP1, hence preventing Rif1 inhibition of replication initiation. Like yeast Rif1, the dipteran Rif1 homologs have a cluster of conserved CDK and DDK sites near the phosphatase interaction motif in the C-terminal part of the protein (Fig 5A, S3 Fig). This region of Rif1 is also proposed to contain a DNA-binding domain [52][53]. These potential phosphorylation sites cluster in a region of the protein predicted to be of high intrinsic disorder [54] (S3 Fig), a feature associated with phosphorylation events that regulate interactions [55]. Of particular interest are a number of conserved SS/TP sequence motifs. DDK is known to phosphorylate the first serine in SSP motifs only after a priming phosphorylation by CDK on the second serine. Full phosphorylation of such sites is likely to require the dual input of both CDK and DDK. We further examined the role of the more C-terminal potential phosphorylation sites in regulating the late replication activity of Rif1.

To determine if Rif1 is phosphorylated in vivo, we examined Rif1 from 1-h-old embryos, a time when the abundant maternally-provided Rif1 protein would be held inactive by Cdk1. Protein extracts from pooled staged embryos were run on a phos-tag SDS-PAGE gel before or after treatment with lambda phosphatase and subjected to western blotting. All detectable Rif1 exhibited a phosphatase-reversed shift, indicating that most Rif1 is phosphorylated in the pre-MBT embryo (Fig 5B). Comparison of protein samples from pre-MBT and MBT-age embryos revealed that the extent of the shift is somewhat reduced at the MBT stage, consistent with developmentally-regulated reduction in the degree of phosphorylation (Fig 5B).

Two studies in yeast suggested that phosphorylation of Rif1 inhibits its function by blocking interaction with PP1a [37][38], although direct evidence for this idea is lacking, and the prior literature did not define a mechanism by which the repressive action of Rif1 is overcome. *Drosophila* Rif1 has previously been shown to bind to PP1a [40], so we looked for a change to this interaction in conjunction with the developmental down-regulation of Cdk1. As shown above, high Cdk1 activity in early pre-MBT embryos suppresses Rif1 association with chromatin and promotes a short S phase, whereas down-regulation of Cdk1 at the MBT is required for Rif1-mediated extension of S phase. Rif1 was immunoprecipitated out of lysates derived from 20-min collections of embryos aged for either 30 min (pre-MBT) or for 2 h and 15 min (post-MBT). While we expected minimal association of PP1 to Rif1 in pre-MBT extract when Rif1 is robustly phosphorylated, western blotting showed a robust PP1 signal (Fig 5E). Additionally, Rif1 immunoprecipitated from the post-MBT extract was accompanied by a similar amount of PP1. Hence, the interaction between Rif1 and PP1a, at least at bulk levels, is not regulated in a way that explains the onset of late replication, and Rif1 appears to interact effectively with PP1 at early stages when it is phosphorylated and held inactive by Cdk1 kinase.

To further explore possible regulation of Rif1 by phosphorylation, we mutated candidate phospho-sites. We selected 15 S/T residues within CDK or DDK consensus motifs located in the C-terminus of Rif1 and mutated them to alanine (Fig 5A) to prevent their phosphorylation. We reasoned that this phospho-site mutant Rif1, hereafter referred to as Rif1.15a, might not be inhibited by S phase kinases and so act as a dominant gain-of-function allele. Ectopic expression of Rif1.15a during the blastoderm divisions by injection of in vitro-transcribed mRNA causes extensive anaphase bridging as typically seen when DNA replication is incomplete (Fig 5C). We never observed this effect after injection of mRNA encoding wild-type Rif1, arguing that this is not a simple overproduction phenotype. To test if the gain-of-function Rif1 mutant still operated through its associated phosphatase, we mutated the conserved PP1 interaction motif RVSF in the Rif1.15a construct. Ectopic expression of the resulting Rif1.15a-RaSa mutant did not disrupt cell cycle progression (Fig 5C). In addition, we generated transgenes expressing either wild-type Rif1 or Rif1.15a under upstream activating sequence (UAS) control. Overexpression of Rif1 in the eye imaginal disc (and more weakly throughout much of the head capsule) using the eyeless-Gal4 driver did not disturb eye formation, whereas expression of Rif1.15a caused complete lethality with pupae developing into headless nearly adult flies (Fig 5D). The severity of this phenotype suggests that expression of Rif1.15a disrupted the early proliferative period of the eye-antennal disc. These assays suggest that Rif1.15a has a damaging gain-of-function action, as might be expected if it were immune to regulation of its ability to inhibit DNA replication.

Because our data indicated that the chromatin binding of Rif1 was regulated during S phase by Cdk1 activity, we examined the influence of mutation of the phospho-sites on Rif1 localization. Using mRNA injection, we expressed a GFP-tagged version of Rif1.15a and followed its localization and the consequence of its expression in live records. Embryos were injected with Rif1.15a-GFP mRNA during cycle 12. The timing of this injection allowed sufficient fluorescent protein to accumulate so that its behavior could be followed throughout cell cycle 14, while still minimizing the damage due to induced catastrophic mitoses in the earlier cycles. Rif1.15a was recruited to specific chromatin foci normally at the start of S phase, indicating that the mutated residues are not required for the binding specificity of Rif1. Imaging Rif1.15a-GFP throughout cycle 14 yielded several interesting results that were never observed when imaging endogenous Rif1-GFP or ectopic wild-type Rif1-GFP expressed by mRNA injection. Unlike the dynamics of the wild-type protein, Rif1.15a continued to accumulate on heterochromatin throughout most of S phase 14. It then showed a slow decline but remained bound in the following G2 and into mitosis 14 (Fig 5F). The Rif1.15a foci on newly condensed mitotic chromosomes were localized to pericentric regions, where the satellites reside. On progression into anaphase, bridges were seen connecting the separating chromosomes, and Rif1.15a-GFP specifically labeled these chromatin bridges (Fig 5F inset). As bridged nuclei exited mitosis, an unbound pool of Rif1.15a was recruited to the chromocenter, presumably after new pre-RCs were loaded. This abrupt recruitment of Rif1.15a shows that mutation of the selected sites did not fully eliminate cell cycle–regulated behavior of Rif1. Nonetheless, the dramatic consequence of the phosopho-site mutations shows that these sites contribute importantly to Rif1 dissociation from chromatin and that in the absence of dissociation Rif1.15a is capable of blocking replication to give anaphase bridging when chromosomes are driven into mitosis. In contrast to previous suggested mechanisms, we conclude that the repressive action of Rif1 on DNA replication is regulated by kinase-driven dissociation from chromatin.

### Rif1 is dispensable for survival and fertility

A recent study reported that Rif1 was an essential gene in *Drosophila* based on partial lethality of a ubiquitously expressed RNAi against Rif1 [40]. We created a precise deletion of the Rif1 open reading frame (ORF) using CRISPR-associated protein 9 (CRISPR-Cas9) (S4 Fig). While the mutation does cause reduction in survival, we find that the homozygous *rif1* null gives viable, reproductively competent flies that can be propagated as a stock. Zygotic loss-of-function *rif1* mutants develop to adulthood with a reduced survival and a male-to-female ratio of 0.4 (*n* = 189 flies). Additionally, embryos laid by *rif1* mutant mothers (hereafter called *rif1* embryos) have a reduced hatch rate (55% of control, *n* = 500 embryos). We conclude that Rif1 is dispensable in *Drosophila*, at least in our genetic background, and we suspect that the previously reported lethality and sterility included enhancement of the loss-of-function phenotype by off-target effects of the RNAi. Though surprised by the viability of the Rif1 deletion, it provided an opportunity to examine the phenotype of complete absence of function.

### Rif1 is required for the onset of late replication and the prolongation of S phase at the MBT

**Fig 6:**
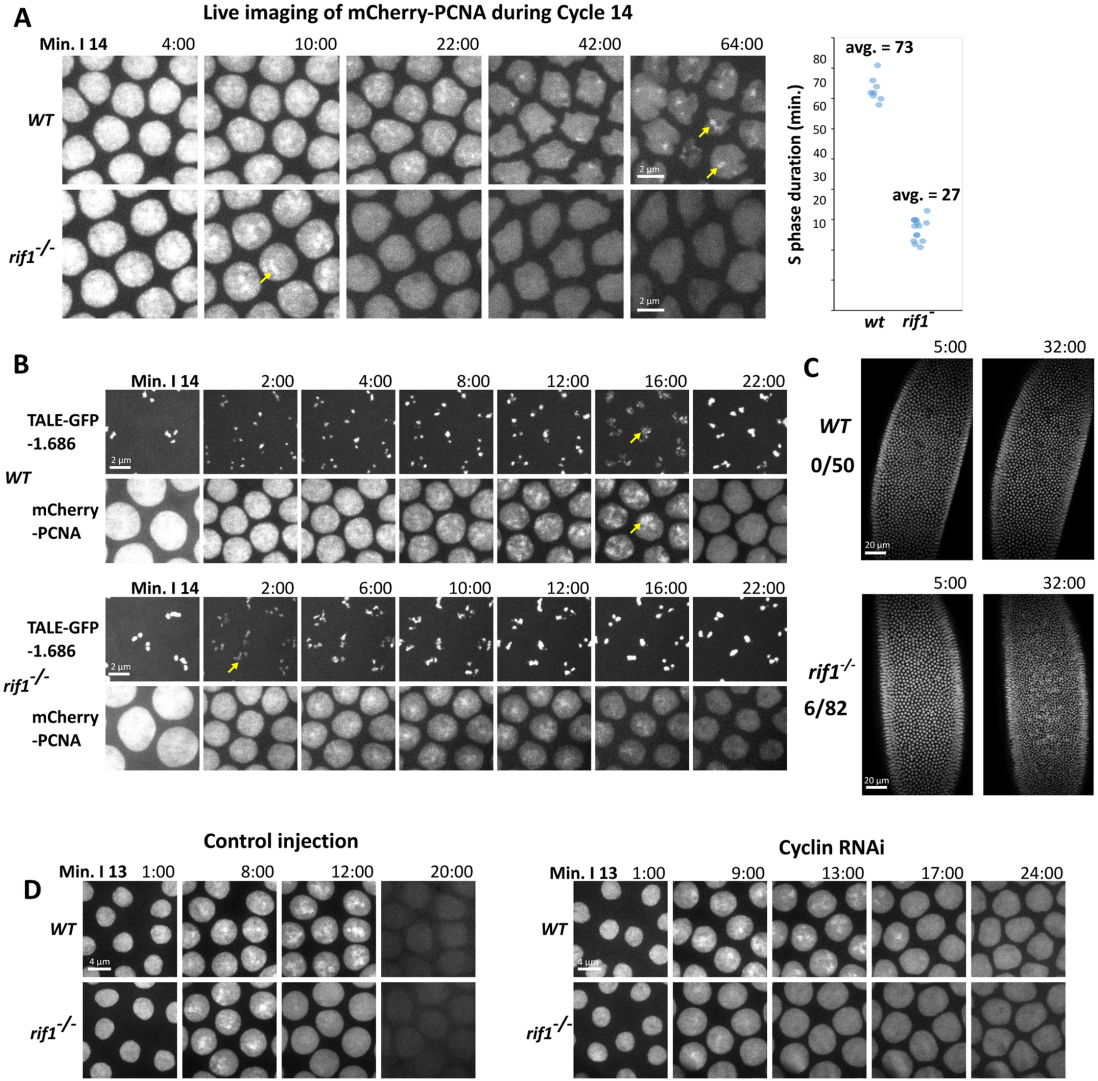
*rif1* is required for the prolongation of S phase at the MBT. (A) Stills from time-lapse imaging of mCherry-PCNA during S phase 14 in WT (top) or *rif1* mutant (bottom) embryos. Yellow arrows indicate late replication foci. The plot on the right displays the total duration of S phase 14 in WT and *rif1* embryos (S1 Data). Each data point represents a distinct embryo. The duration of S phase was scored from real-time records of PCNA dynamics. (B) Stills from time-lapse imaging following the TALE-light probe for the 1.686 satellite (upper) and mCherry-PCNA to follow replication during S phase 14 in WT and *rif1* embryos. Yellow arrows indicate the decompacted signal from the 1.686 TALE-light during the replication of the 1.686 repeat. (C) WT (top) or *rif1* (bottom) embryos were injected with GFP-HP1a to visualize nuclei and filmed during cycle 14. Of the *rif1* embryos, 6 out of 82 underwent an early mitosis 14 (S8 Movie). (D) Still frames from movies following mCherry-PCNA during S phase 13 in WT and *rif1* embryos after either control or triple cyclin RNAi injection. Mitotic-cyclin knockdown arrests the cell cycle but extends S phase in WT but not *rif1* embryos. GFP, green fluorescent protein; HP1a, heterochromatin protein 1a; MBT, mid-blastula transition; PCNA, proliferating cell nuclear antigen; Rif1, Rap1 interacting factor 1; RNAi, RNA interference; TALE, transcription activator-like effector; WT, wild-type.

Next, we utilized the *rif1*-null mutation to assess the role of Rif1 in the onset of late replication during cycle 14. As discussed previously, real-time records of fluorescent PCNA allow us to follow the progress of S phase and estimate its overall length. In a wild-type S phase 14, PCNA is widely distributed throughout most of the nucleus during the first 10 min but resolves into bright puncta that are obvious for over an hour into interphase, after which only a dim nuclear background is visible. During the final 30 min of S phase, a small number of bright PCNA foci appear and then disappear sequentially as a result of a protracted schedule of late replication (Fig 6A). By using the disappearance of the last PCNA focus as an indication of the completion of replication, we determined that the average S phase 14 lasts for 73 min (Fig 6A). In contrast, S phase 14 in *rif1* embryos from *rif1* mothers was significantly shorter, lasting an average of 27 min. Additionally, there were clear differences in the appearance of the PCNA signal as S phase progressed. The initial period of widespread early replication resolved into PCNA foci, but appearance of these foci was more simultaneous than sequential, and there was no protracted sequence of late foci (Fig 6A). Thus, this S phase 14 resembled the earlier S phases in showing a marginal extension with slightly delayed replication of some foci. However, the normally dramatic extension of S phase 14 is dependent on *rif1*. Failure to extend S phase 14 was observed in all embryos derived from homozygous mothers even though heterozygous fathers were included in the cross. We conclude that maternal Rif1 is required for the normal extension of S phase 14.

Because we had observed that the satellite 1.686 recruited Rif1 during S14, we wondered if its replication time would be altered in *rif1* embryos. To measure the replication time of this repeat, we injected the GFP-1.686 TALE probe into wild-type and *rif1* embryos expressing mcherry-PCNA and recorded S phase 14. We used the transient recruitment of mcherry-PCNA and the obvious decompaction of the marked 1.686 sequences as indictors of active replication of 1.686. In control embryos, the 1.686 repeat began replication 18 min into S14 and completed by 30 min (Fig 6B). In contrast, in *rif1* embryos, 1.686 began replication by 4 min into S14 and completed by 13 min (Fig 6B). We conclude that the 1.686 satellite sequence replicates with a minimal delay in S phase 14 in the absence of Rif1 and that the normally substantial delay requires Rif1.

During the MBT, the embryo degrades both the mRNA and protein of the mitotic activator Cdc25. This allows the introduction of the first embryonic G2 after the completion of the prolonged S phase 14. Mitosis 13 is then the last synchronous division during development, and mitosis 14 relies on the developmentally patterned zygotic expression of new Cdc25. Perturbations that interfere with the down-regulation of Cdk1 can lead to an additional synchronous mitosis. Additionally, since down-regulation of Cdk1 occurs during S phase 14, the embryo can also progress to an additional synchronous mitosis if S phase is eliminated or dramatically shortened, as seen following injection of geminin or alpha-amanitin, respectively [25][26]. We noticed that a small number of *rif1* embryos (7%) executed an early mitosis 14 at approximately 30 min into interphase. The observed mitoses were complete, with no bridged chromosomes, but there was substantial nuclear fall in (Fig 6C, S8 Movie). We interpret the incomplete penetrance of the extra-division phenotype to be an indication that the duration of S phase in *rif1*-null embryos is close to a threshold so that, in most embryos, the cyclin:Cdk1 activity declines enough to introduce a G2, but in some embryos, the residual maternal cyclin:Cdk1 function remains high enough to trigger mitosis upon the completion of the shorten S phase. The result shows that Rif1 is important for reliable coordination of the MBT.

Previous work has shown that before the MBT, Cdk1 is required for driving a short S phase. Our finding that Cdk1 activity suppresses Rif1 function in cycle 14 (Fig 4) suggests an explanation for the early role of Cdk1 in promoting short S phases; interphase activity of Cdk1 prevents Rif1 from prematurely introducing late replication. This model predicts that reducing Cdk1 activity would not be able to prolong a pre-MBT S phase in the absence of *rif1*. To test this idea, we examined S phase 13 in wild-type or *rif1* embryos after knockdown of mitotic cyclins, essential activators of Cdk1, by RNAi. Wild-type control–injected embryos exhibit transient but obvious foci of PCNA staining during late S phase 13 and replicate their satellite sequences with a slight delay in this S phase. While *rif1* embryos still exhibit PCNA foci, these foci are even shorter lived (Fig 6D). Following injection of mitotic-cyclin RNAi, wild-type embryos increased the duration of S phase 13 from 14 min to 20 min. In contrast, *rif1* embryos did not extend S phase after cyclin knock down (Fig 6D). This demonstrates that the requirement for Cdk1 in the timely completion of S phase 13 can be bypassed by loss of *rif1*. However, S phase 13 in the *rif1* mutant embryos is still longer than very early embryonic S phases, which can be as short as 3.4 min. Thus, Cdk1 down-regulation of Rif1 contributes to S phase prolongation in cycle 13, but there must be additional factors influencing the progressive prolongation of early cycles.

### *Cdc7* is essential during the early embryonic S phases, and the requirement can be substantially bypassed by removal of *rif1*

The cell division cycle 7 (Cdc7)-Dbf4 kinase complex (or DDK) is required for origin initiation in many systems. In *S*. *cerevisiae*, the essential function of DDK is thought to be the phosphorylation of the MCM helicase complex during pre-RC activation [16]. In both *S*. *pombe* and *S*. *cerevisiae*, the deletion of *rif1* partially rescues S phase and viability in *cdc7* mutants. Two functional interactions have been proposed to contribute to this finding. DDK appears to phosphorylate and inactivate Rif1, thereby activating replication by a derepression input. Indeed, the presence of conserved DDK phosphorylation motifs in Rif1 suggests that DDK may synergize with CDK in the inactivation of Rif1. This input would be dispensable in a *rif1* mutant. Additionally, Rif1 is thought to inhibit replication by recruiting PP1 to the origin and dephosphorylating the MCM helicase, an action that opposes DDK. The loss of this opposing activity in a *rif1* mutant might reduce but would eliminate the DDK activity required for MCM phosphorylation. We wanted to assess the possible parallels in *Drosophila* to clarify the involvement of Rif1 in replication control.

Despite strong conservation, *cdc7* has not been well studied in *Drosophila*. In *Drosophila*, *cdc7* is an essential gene, and recent work has demonstrated that in complex with the *dbf4* ortholog *chiffon*, *Drosophila* Cdc7 can phosphorylate Mcm2 in vitro and that *cdc7* is required for endocycle S phases in follicle cells [56]. However, the function of DDK during mitotic S phase has not been described.

**Fig 7:**
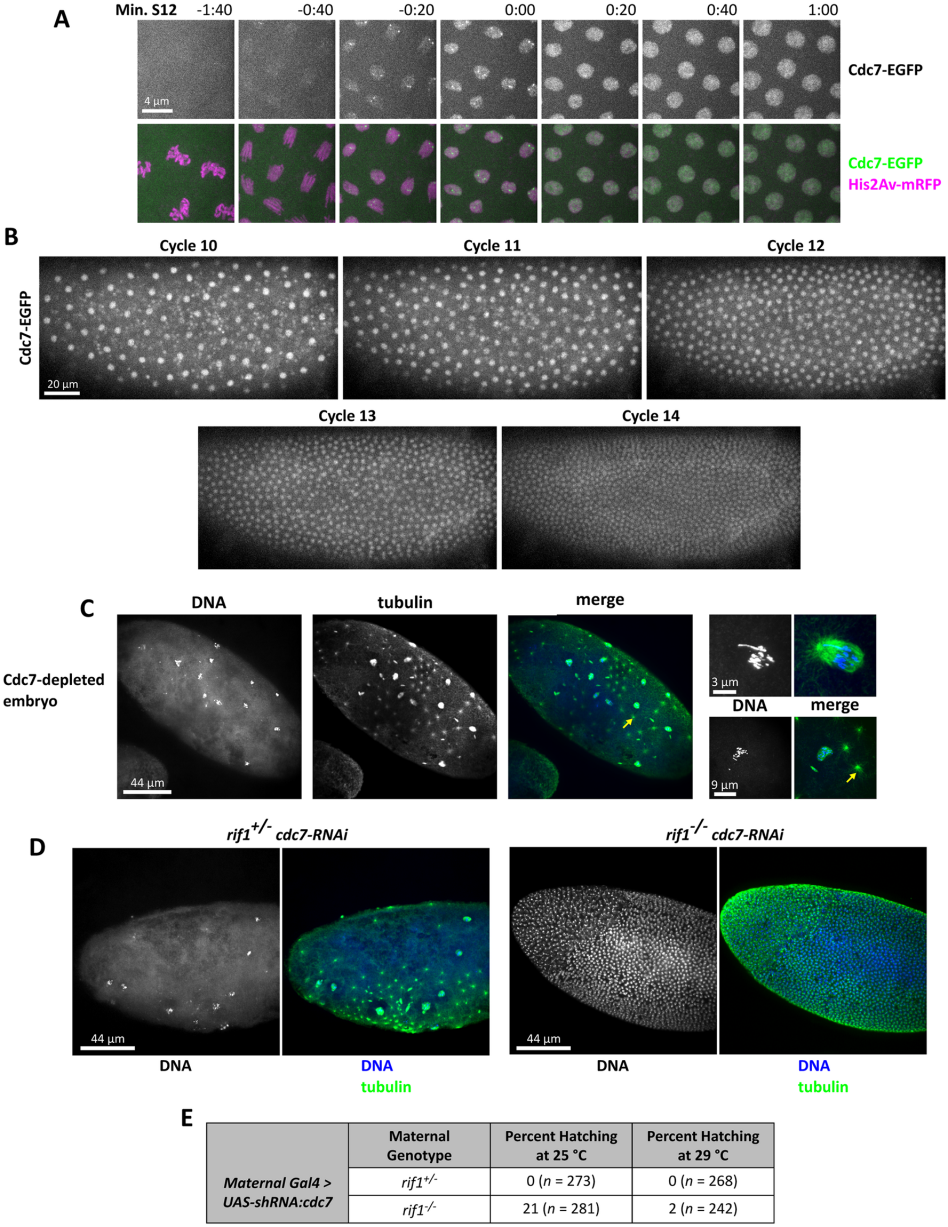
Deletion of *rif1* rescues cell cycles to Cdc7-depleted embryos. (A) Cell cycle–regulated recruitment of Cdc7 to the nucleus during early interphase shown by time-lapse microscopy of Cdc7-GFP, His2Av-RFP during nuclear cycle 12 using a 100× objective. (B) Live imaging of Cdc7-GFP embryos during cycles 10–14 reveals dilution of nuclear Cdc7 over the pre-MBT divisions (S9 Movie). (C) Fixed Cdc7-depleted embryos stained for DNA and tubulin. Yellow arrow indicates a centrosome not associated with a DNA mass. Adjacent enlarged images display magnified views of abnormal mitotic structures. (D) Fixed *rif1* mutant embryo after maternal RNAi against *cdc7* stained for DNA and tubulin. Deletion of *rif1* restores cell cycles and early development to Cdc7-depleted embryos. (E) Hatch rate of embryos laid from mothers either heterozygous or homozygous for a deletion of *rif1* and expressing maternal RNAi against *cdc7*. The total number of embryos counted is indicated in parentheses. The temperature at which both the flies were reared and the hatch rate performed is indicated. Cdc7, cell division cycle 7; EGFP, enhanced green fluorescent protein; GFP, green fluorescent protein; His2Av, histone 2A variant; MBT, mid-blastula transition; mRFP, monomeric red fluorescent protein; RFP, red fluorescent protein; Rif1, Rap1 interacting protein 1; RNAi, RNA interference; shRNA, short hairpin RNA; UAS, upstream activating sequence.

To address this issue, we first tagged endogenous *cdc7* with GFP using CRISPR-Cas9. The resulting *cdc7-GFP* stock was healthy and fertile, indicating that the tag did not disrupt the essential function of Cdc7. Time-lapse imaging of Cdc7-GFP embryos during syncytial development revealed that Cdc7 localization was cell-cycle regulated. Cdc7 was nuclear during interphase, dispersed into the cytosol during mitosis, and was rapidly recruited to late-anaphase chromosomes and concentrated in the telophase nucleus (Fig 7A). Initial Cdc7 recruitment featured two transient bright foci followed by fine puncta, suggesting that it is recruited to chromatin at the time at which pre-RCs are undergoing activation during the syncytial cell cycles. Comparison of nuclear Cdc7 at equivalent times in subsequent cell cycles (early S phase) leading up to the MBT revealed a decline in the per-nucleus protein signal. Since nuclear size decreases with each division, if the amount of Cdc7-GFP in each nucleus were constant throughout these cycles, then the local intensity (concentration) of nuclear fluorescence would increase with each division. Instead, brightness decreases. This observation is consistent with subdivision of limited and/or declining pool of Cdc7 among an increasing number of nuclei. If level were limiting, this would result in diminishing availability of Cdc7 to fire origins in later cycles (Fig 7B, S9 Movie).

To determine if *cdc7* is required for DNA replication during the early cell cycles, we depleted DDK from the embryo using maternal-tubulin Gal4 to drive RNAi against *cdc7* during oogenesis. This setup did not interfere with egg production, although we found that expression of RNAi against *cdc7* using the earlier acting maternal triple driver (MTD)-Gal4 did cause severe defects in egg morphology, indicating that *cdc7* does play a role in the female germline. Embryos depleted of Cdc7 using maternal-tubulin Gal4 failed to hatch. Cytological examination of Cdc7-depleted embryos revealed penetrant defects during the early preblastoderm divisions. We observed multiple highly fragmented DNA masses that were unevenly distributed in the embryo interior, along with scattered abnormal mitotic structures (Fig 7C). In all cases, nuclei failed to form a blastoderm, although in many cases, centrosomes appeared to continue to duplicate in the absence of any associated DNA (Fig 7C). Such a dissociation of the embryonic nuclear and centrosome cycles has been described before in embryos injected with the DNA polymerase inhibitor aphidocolin during the preblastoderm cycles [57]. We conclude that Cdc7 is essential for effective nuclear cycles in the early embryo, consistent with a requirement in DNA replication.

Next, we tested for a genetic interaction by depleting Cdc7 from *rif1* mutant embryos using maternally expressed RNAi at 29 °C. In the absence of *rif1*, 2% (*n* = 242) of Cdc7-depleted embryos hatched, while Cdc7-depleted embryos from mothers heterozygous for *rif1* (*rif1*^*+*^) never hatched (Fig 7E). Cytological examination demonstrated that removal of *rif1* restored cell cycle progression to Cdc7-depleted embryos. *rif1* mutants completed substantially more cell cycles than heterozygous control embryos, with most progressing to the blastoderm stage. Many of the blastoderm-rescued embryos showed abnormalities such as substantial nuclear fall in, disorganized nuclear spacing, and nonuniform nuclear density. Nonetheless, some of the observed embryos attempted cellularization and gastrulation (Fig 7D), with the rare cases of hatching indicating occasional success. Reducing the temperature to 25 °C, thereby reducing the activity of the Gal4 protein driving the RNAi, improved the hatch rate of *rif1* mutant embryos to 21% (*n* = 281) while still causing 100% lethality in embryos from *rif1* heterozygous mothers (Fig 7E). We conclude that deletion of *rif1* can restore embryonic cell cycle progression and early development to Cdc7-depleted embryos. However, this rescue is incomplete, likely because of altered regulation of the restored S phase in such doubly defective embryos.

### The Rif1-regulated onset of late replication precedes the establishment of heterochromatic marks

Early embryonic chromatin lacks specializations that come to distinguish different regions of the genome at later stages. Thus, the order of appearance of different specializations can give us insight into the hierarchy of regulation. Since late replication is considered a feature of heterochromatin, we expected its emergence to follow the embryonic appearance of the hallmarks of heterochromatin. Our recent work described the onset of localized H3K9me2/3 and HP1a [30]. While a very low level of modification and chromatin-bound HP1a was detected before the MBT, a period of abrupt accumulation of HP1a and more extensive H3K9me2/3 modification only occurred later, during S phase 14. Here, we explore the relationship between this HP1a-centered program and the action of Rif1 in the control of late replication.

**Fig 8:**
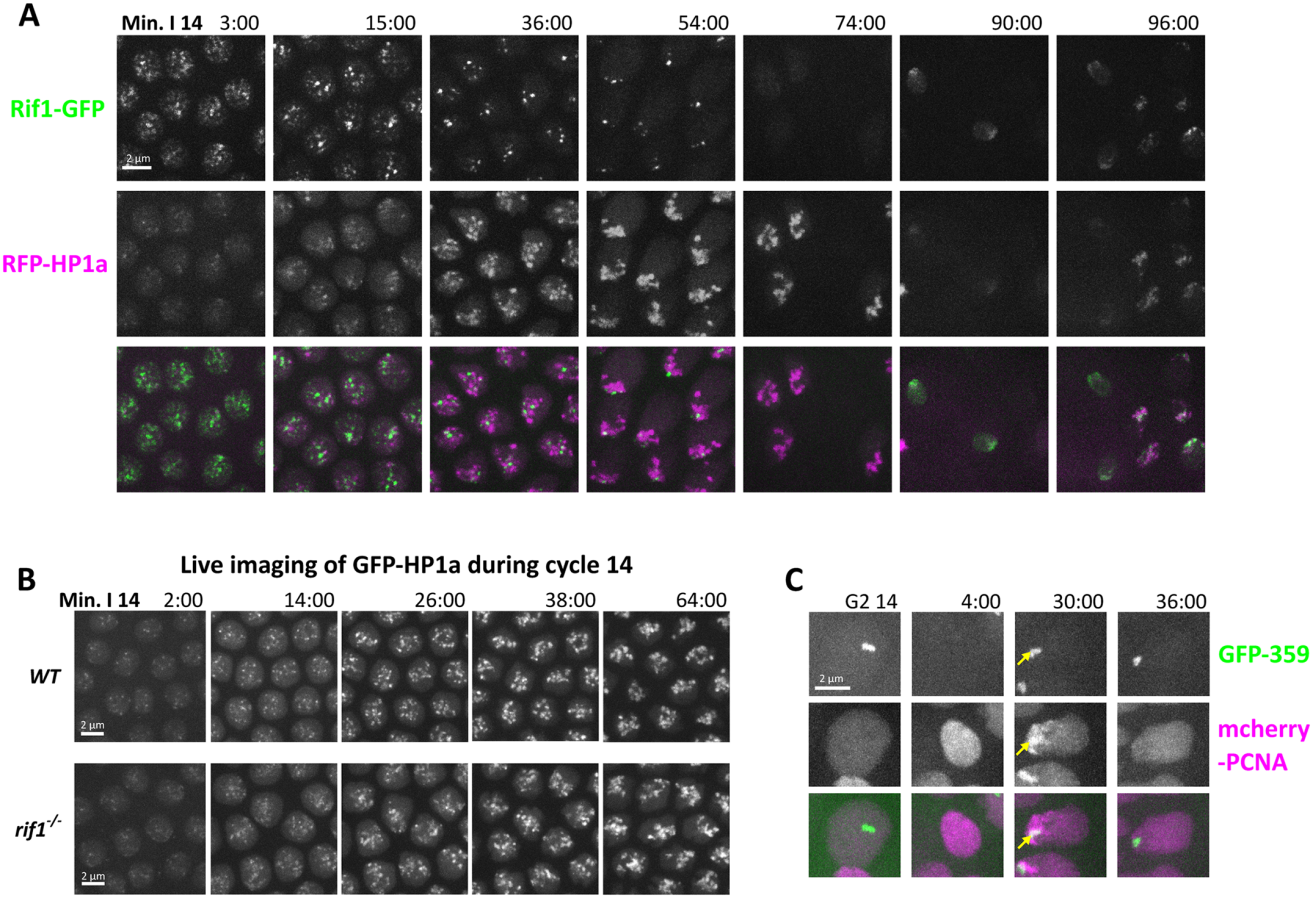
Sequential appearance at satellite sequences suggests that Rif1 influences late replication prior to an impact of HP1a. (A) Still images from time-lapse imaging of Rif1-GFP and RFP-HP1a during cycle 14 (early S phase through mitosis). During S phase, the number of Rif1 foci decline as the number of HP1a foci increase. G2 nuclei lack Rif1 foci but retain strong HP1a foci (74 min). During the asynchronous mitosis 14 (74/90/96 min), both proteins are lost but are rapidly recruited to late anaphase chromosomes (90/96 min). (B) Still images from time-lapse imaging of GFP-HP1a protein injected into WT embryos (above) and *rif1* embryos (below) during S phase 14. HP1a recruitment to the heterochromatin proceeded similarly in control and mutant embryos. (C) Still images from time-lapse imaging of the replication of the 359-satellite progressing from G2 of cycle 14 until completion of its replication in cycle 15. Note that the TALE-light signal is not immediately visible following mitosis (4:00 min frame). We previously described how HP1a binds to the 359 bp repeat following its replication during S phase 14 and subsequently delays its replication in S phase 15. In *rif1* embryos, the 359 repeat also replicated late in S phase 15 (in frame 30:00, yellow arrow indicates where the 359 TALE signal overlapped with PCNA). GFP, green fluorescent protein; HP1a, heterochromatin protein 1a; PCNA, proliferating cell nuclear antigen; RFP, red fluorescent protein; Rif1, Rap1 interacting factor 1; TALE, transcription activator-like effector; WT, wild-type.

During cycle 14, Rif1-GFP and HP1a-red fluorescent protein (RFP) bound to localized foci in the same position but never at the same time. Rif1 foci disappear sequentially during cycle 14, while HP1a is diffusely localized in early cycle 14 and is subsequently recruited to apical foci that multiply and intensify. Importantly, in live imaging of Rif1-GFP and HP1-RFP, we do not observe any overlap between the two signals in S14 (Fig 8A). This finding is in accord with our finding that PCNA foci and Rif1 do not overlap (Fig 2A) and our previous demonstration that PCNA and HP1 foci do not overlap in cycle 14 [26]. Together with the timing of recruitment of these proteins to specific satellites, these observations show a sequence in which satellites lose associated Rif1 before they initiate replication, as marked by PCNA, and then complete replication before binding HP1. We conclude that the introduction of late replication by Rif1 precedes the binding of HP1a in cycle 14. In contrast, at the start of cycle 15, both Rif1 and HP1a are rapidly recruited to the chromocenter, with Rif1 binding slightly earlier (Figs 2B and 3B), and we have previously detected an influence of HP1 on replication timing in this cycle [30].

Given the earlier binding of Rif1 to satellite sequences in cycle 14, Rif1 might direct subsequent heterochromatin formation. In fission yeast, Rif1 is required for the maintenance of silencing at some heterochromatic sites in the genome [58]. We examined whether Rif1 influences the emergence of HP1a-bound heterochromatin. GFP-HP1a was injected into either wild-type or *rif1*-null embryos and imaged during cycle 14. We observed no difference between HP1a recruitment between the two genotypes, indicating that HP1a binds independently of Rif1 in the fly embryo (Fig 8B).

We had previously found that the recruitment of HP1a to the 359 bp–repeat satellite sequence during cycle 14 occurred only after its replication and was unimportant to the replication timing of this satellite in S phase 14. However, this recruitment of HP1a was required for a shift of 359 replication to a much later time in S phase of cycle 15 [30]. Because our results demonstrate that the recruitment of HP1a to the heterochromatin during S phase 14 is independent of Rif1, we wondered if the replication time of 359 was altered in *rif1* embryos in cycle 15. Live imaging of mCherry-PCNA-expressing *rif1* embryos injected with GFP-359 TALE probes indicated that 359 still replicates late during S15 (Fig 8C). We conclude that an HP1a-dependent program can delay replication of 359 sequences in cycle 15 without Rif1 input. It seems likely that this HP1a-dependent program operates to influence the replication of many heterochromatic sequences after cell cycle 14.

## Discussion

We studied the onset of late replication in its natural setting during early embryogenesis. We evaded complexities that are added later by this focus on the first delays in replication of particular sequences. We achieved three things of importance. We show how S phase is dramatically extended at the embryonic MBT by Rif1-mediated temporal programming of replication. We show that this temporal program does not rely on other features of heterochromatin, such as HP1 binding, that are only introduced later in development. We detail precise coordination of Rif1 dissociation from chromatin foci with the onset of their replication. Together, our findings suggest that an interplay of Rif1 and S phase kinases governs developmental prolongation of S phase at the MBT and underlies a timer of the replication program in this first extended S phase.

One of our central findings is that Rif1 function is responsible for the onset of the late-replication program in cell cycle 14. Since Rif1 is provided maternally and is present at high levels during the earlier rapid cycles, something must be limiting its action prior to the MBT. Previous work showed that timely completion of the pre-MBT S phases requires persistent Cdk1 activity. We found that Cdk1 activity promotes dissociation of Rif1 from chromatin and inhibits its function as a replication inhibitor and that the requirement for high Cdk1 for a rapid early S phase was eliminated in Rif1 mutant. Thus, Cdk1 activity suppresses pre-MBT function of Rif1 to promote early rapid S phases. But Cdk1 does not appear to act by itself in suppressing early Rif1 function. While early embryonic cycles were arrested by depletion of Cdc7, loss of Rif1 suppressed this defect, arguing that a major role of Cdc7 is to promote rapid cell cycles in the presence of maternal Rif1. Consequently, we suggest that both CDK and DDK kinases are critical to prevent preloaded Rif1 from interfering with rapid S phase completion in early cycles. Indeed, SS/TP motifs in the C-terminus of Rif1, whose full phosphorylation is expected to require priming by CDK followed by DDK action, are conserved among distantly related insects (S3 Fig). We suggest that collaboration between CDK and DDK is essential for the full regulation of Rif1. After the MBT down-regulation of Cdk1, DDK may function with Cdk2, but together, they are less proficient at driving Rif1 dissociation from late origins.

**Fig 9:**
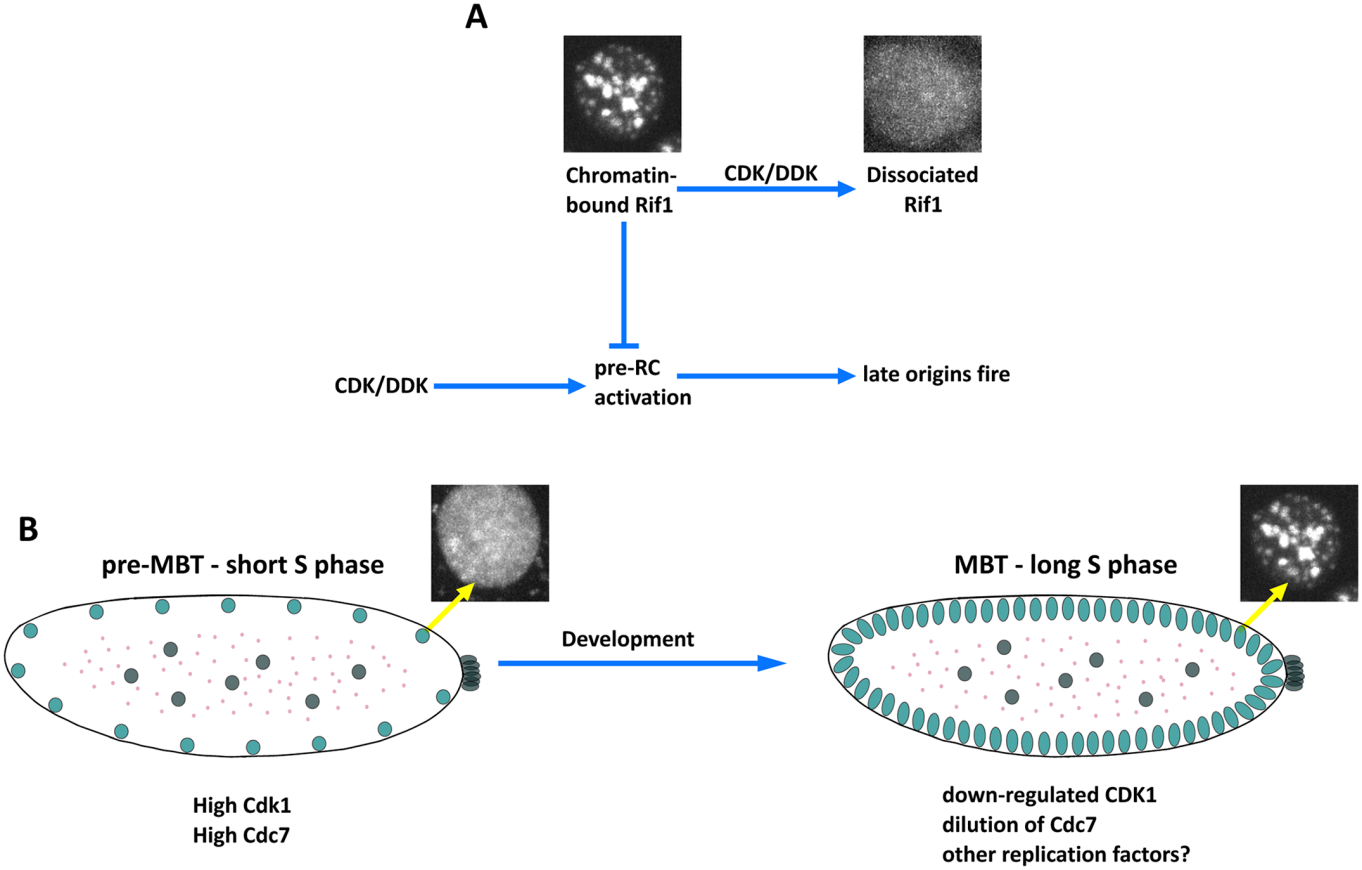
(A) Interactions of the S phase kinases and the Rif1 inhibitor create a late replication program. In a nucleus in early S phase (left), foci of compacted late replicating satellites have bound Rif1 that creates a phosphatase-rich domain through its interaction with PP1a. This protects the pre-RC from the activating influence of S phase kinases CDK and DDK. With time (to the right), the S phase kinases act on Rif1, first gradually, to promote its dissociation, and then abruptly as the kinases overwhelm the weakened domain of phosphatase dominance. At this time, the S phase kinases can successfully directly target and activate the pre-RC. (B) During the early pre-MBT cycles, high CDK and DDK activities rapidly drive Rif1 dissociation so that delays in replication are initially negligible. A progressive lengthening of the embryonic S phase is associated with incrementally increasing delays in satellite replication. Part of this delay is due to declining CDK and increasing, but still transient, Rif1 association. However, Rif1-independent factors, likely the titration of several replication proteins such as Cdc7, also contribute to this slowing. Then, at the MBT, developmentally programmed down-regulation of Cdk1 allows persistent binding of Rif1 to satellite DNA and the introduction of a late replication program, thereby extending the duration of S phase. Cdc7, cell division cycle 7; CDK, cyclin-dependent kinase; Cdk1, cyclin-dependent kinase 1; DDK, Dbf4-dependent kinase; MBT, mid-blastula transition; PP1a, protein phosphate 1a; pre-RC, pre-replicative complex; Rif1, Rap1 interacting factor 1.

Importantly, the ability of Cdk1 to limit Rif1 function provides a key link in the developmental control of the MBT. A developmental decline in Cdk1 triggers the extension of the cell cycle at the MBT [27][28]. However, it was not previously apparent how a decline in Cdk1, a mitotic kinase, would trigger the abrupt extension of S phase that marks the onset of cell cycle slowing at the MBT. We show that decline of Cdk1 activity is necessary and sufficient to release constraints on the pool of Rif1, which then acts to extend the S phase 14 (summarized in Fig 9). Unlike other cell cycle transitions, which are associated with marked regulatory changes, the end of S phase is defined simply by the completion of replication. Because of a progressive reduction in the amount of DNA undergoing replication as S phase progresses, assessments of the very end of replication are expected to vary depending on sensitivity as well as methods of replication detection and timing. The real-time observation method that we have used here defines the end of S phase as the time of dissipation of the last PCNA focus. It was previously validated as a measure of S phase length in studies comparing incorporation of injected fluorescent deoxy-triphosphates to the progressive dispersal of fluorescent PCNA foci in the *Drosophila* embryo [26]. We show that Rif1 binds the last-detected focus of replication until it begins its much-delayed replication during S phase 14. This long S phase is then followed by the first embryonic G2 and a mitosis that is triggered by patterned zygotic transcription of Cdc25.

Embryos provide a unique situation in which to study the role of time in biology, because development progresses through a stereotyped sequence of events. Our work takes advantage of this in two ways. First, because events that occur early cannot be caused by events that happen only later, our description of the sequential emergence of the different features and behaviors that characterize heterochromatin tests the interdependence of these features. During the rapid pre-MBT cell cycles, the satellite DNA is already compacted prior to appearance of other heterochromatic features [26]. Upon Cdk1 down-regulation at the MBT, we now show that Rif1 binds to the satellite sequences and delays their replication, marking onset of late replication in cycle 14. Satellite-repeat sequences only begin to accumulate obvious HP1a after their replication in cycle 14, on time to make a contribution to delayed replication in later cycles but not during S phase 14 [26, 30]. Second, embryos provide us with precision. By following a natural late-replication program in a specific cell cycle using live imaging, we achieve high temporal resolution. During the first post-MBT S phase, different satellite sequences replicate according to a stereotyped schedule [26]; we now detail precise coordination of Rif1 dissociation from chromatin foci with the onset of their replication. We further show that Rif1 is required for this temporal program and that a mutant of Rif1 that resists dissociation blocks replication. Finally, the impact of CDK/DDK on Rif1 suggests that interplay of Rif1 and S phase kinases underlies a timer for a late-replication program.

### Isolating a function of Rif1 as a developmental regulator of the replication timing program

Rif1 is widely conserved, and it has been implicated in several functions—notably, the control of telomere growth, replication timing, and DNA damage responses [35]. Despite this complexity, the association of Rif1 with replication timing in our studies appears to be uncomplicated by other actions of Rif1. Two factors are likely to contribute to this. First, it is not clear that Rif1 serves the same spectrum of functions in all of the organisms in which it is found. For example, the distinctive telomeres of *Drosophila* do not appear to be regulated by Rif1 [52]. Additionally, previous tests of the function of *Drosophil*a Rif1 in cultured cells and when ectopically expressed in other species suggested that *Drosophila* Rif1 protein might lack some functions attributed to the Rif1 of other species [52]. Second, even if the *Drosophila* Rif1 protein has additional functions at other stages in the life of the fly, the first time Rif1 functions in embryogenesis, its role appears to be to impose a replication timing program on S phase. Thus, this developmental context can isolate the replication timing role of Rif1 from other potential functions.

### Programmed interaction of Rif1 with satellite sequences guides their timing of replication

#### Developmentally programmed association of Rif1 with satellite sequences marks the onset of late replication

We have previously characterized the program of replication of satellite sequences. Prior to cell cycle 11, when satellite sequences are early replicating, we did not detect significant recruitment of Rif1 to chromatin. In subsequent pre-MBT cycles, when slight and incrementally increasing delays in satellite sequence replication occur, we see brief association of Rif1 to individual blocks of satellite sequence and an advance in their replication in *rif1* mutant embryos. In pace with the gradual increase in the delay of satellite replication, this Rif1 association persists longer in successive cycles (Fig 1D). In time with the MBT and a dramatic extension of cell cycle 14, we detect more persistent association of Rif1 to satellite sequences.

#### Within each cell cycle, Rif1 dissociation from individual satellite sequences marks the onset of their replication

Individual satellites, each a distinct array of repeats, initiate replication as a unit at a particular time within S phase 14 [26]. The disappearance of different Rif1 foci during S phase parallels the schedule of late replication for the different satellite repeats. Indeed, high-resolution imaging demonstrated that the dissociation of Rif1 is followed closely by the recruitment of PCNA and replication. In addition, TALE-light probes against the satellite 1.686 allowed us to visualize the association of Rif1 with this repeat during S phase. Rif1 staining overlapped that of the satellite early in S phase, and Rif1 dissociated in mid-S phase just before the decompaction and replication of the underlying DNA (Fig 2). Finally, expression of the Rif1.15a gain-of-function mutant blocked dissociation of Rif1 and prevented complete replication (Fig 5). Thus, the dissociation of Rif1 coincides with and is needed for onset of replication.

#### Phosphorylation controls the association of Rif1 with late-replicating chromatin

The control of pre-RC activation by the kinases CDK and DDK appears to be widely conserved among eukarya [59]. The 2 kinase types act by parallel mechanisms phosphorylating their respective target sites in the N-terminal regions of MCM2 and MCM4 to promote activation of the pre-RC. Activation usually requires collaboration of the two kinases. These kinases are active from the outset of S phase, suggesting that the timing program of replication within S phase involves local regulation of their action. Rif1 has emerged as a key regulator of this same step, and it appears to have 2 types of interactions with the activating kinases. Rif1 inhibits and delays replication initiation by recruiting PP1 to the genome where its ability to dephosphorylate kinase targets counters the action of DDK and CDK. Additionally, CDK—and likely DDK—inhibit Rif1, resulting in derepression that further promotes pre-RC activation in a feed-forward action (Figs 5 and 7) [37][38]. We document the opposing action of Rif1 and DDK in the early fly embryo by showing that the Rif1 mutation dramatically suppresses the phenotype of a knockdown of Cdc7, a genetic interaction analogous to those shown in yeasts.

When Cdk1 activity is especially high at metaphase, Rif1 is absent from chromosomes. After metaphase/anaphase inactivation of Cdk1, Rif1 associates with the separating anaphase chromosomes (Fig 3) [34, 40, 46]. Manipulations and mutations that promote Cdk1 activity accelerate dissipation of Rif1 from chromatin (Figs 3D and 4). Reciprocally, inhibition of Cdk1 during pre-MBT S phases increases the amount of time Rif1 spends in foci. Additionally, Rif1 has conserved clusters of Cdk1 target sites, and we observed increased phosphorylation of Rif1 during the early cycles in which Cdk1 activity is high and when Rif1 shows minimal association with chromatin. Finally, mutation of 15 CDK and DDK consensus sites in the C-terminus of Rif1 largely prevented dissociation of Rif1 from chromatin and blocked completion of DNA replication. Our finding that Rif1 mutation restored cell cycle progression to embryos blocked by knockdown of DDK (Cdc7) supports a role for DDK in the suppression of destructive Rif1 activity in early embryos. Thus, both types of S phase–activating kinase suppress Rif1 activity, a coherent feed-forward input that supports direct action of these kinases to activate the pre-RC (Fig 9).

In budding and fission yeast, it was suggested that CDK and DDK inhibit Rif1 by promoting release of PP1 [37][38]. *Drosophila* Rif1 also interacts with PP1a, but we have not detected a change in this interaction when comparing 2 stages, one in which high Cdk1 prevents Rif1 function and another stage when Cdk1 is inactive (Fig 5). Instead, we see regulation in the association of Rif1 to chromatin. The addition of ectopic Cdk1 activity during S phase 14 drove rapid dissociation of Rif1 from chromatin and completion of late replication without delay (Fig 4B, S7 Movie). Although we cannot exclude regulation of phosphatase binding, our results demonstrate a key role for phosphorylation-regulated chromatin binding of the *Drosophila* Rif1. Perhaps both mechanisms operate. Since the regulatory architecture is the same, different organisms might emphasize different means of Rif1 inhibition.

The observed program of Rif1 dissociation suggests that the process could play a role in a mysterious feature of replication timing. At least in higher organisms, large domains of the genome replicate as temporal units at a specific time during S phase, a phenomenon that requires coordinate firing of many linked origins [60]. Concerted dissociation of Rif1 from each satellite sequence is followed by the recruitment of PCNA to the entire domain, suggesting that behavior of Rif1 might coordinate the firing of the numerous origins within each large block of repeating sequence. How might region-specific dissociation of Rif1 itself be coordinated? Our data suggest that dissociation is promoted by phosphorylation of Rif1 (Fig 9). Since Rif1 is known to recruit PP1, the localized phosphatase could counter the action of the kinases and protect Rif1 from dissociation. This antagonism between kinase activity and localized PP1 could create a bistable circuit. Domains enriched in Rif1 would also be enriched in PP1, which could locally protect Rif1 from the kinases. However, even a slow rate of Rif1 release would eventually give the kinases the upper hand within the domain. At the tipping point, dissociation of Rif1 would accelerate. Since our findings show coordinate release of Rif1 from large domains of the genome and show that Rif1 release is coupled to onset of replication, we suggest that the circuit regulating Rif1 release functions both as timer and as a means of coordinating the firing of origins in local domains.

### Rif1-independent contributions to S phase duration

In *rif1* embryos, the early 3.4 min S phase is extended to 27 min by cycle 14. Although much shorter than the 72-min S phase 14 in wild-type embryos, this *rif1*-independent extension of S phase resembled progression normally seen in the earlier blastoderm cycles (11–13). A long-standing suggestion for embryonic slowing of the cell cycle is that the increasing number of nuclei titrate factors required for the speedy early S phases. Several factors might limit pre-RC activation. For example, Cdc7 has an input as demonstrated by its genetic interaction with Rif1. Given that we saw diminishing concentration of nuclear Cdc7 as it was distributed to an increasing large number of nuclei over successive divisions, it is possible that developmental declines in this activating input could contribute to slowing of S phase, although at present, we do not have evidence demonstrating that this decline is of regulatory importance. Notably, the concentrations of several proteins that are recruited to the pre-RC during its maturation influence the speed and efficiency of pre-RC activation in yeast and in *Xenopus* [24][61].

Investigations of S phase extension occurring at the *Xenopus* MBT have emphasized multiple inputs influencing activation of the pre-RC [61][62]. A recent analysis suggested that Dbf4 related factor 1 (Drf1), the activating subunit for the Cdc7 kinase in the *Xenopus* embryo, is subject to active destruction at the MBT [63]. Failure to down-regulate DDK sensitized embryos to the further stress of overproduction of 3 replication factors that promote pre-RC maturation. Indeed, the authors suggest that titration of the maternal supply of these 3 replication proteins cooperate with active destruction of Drf1 to slow S phase at the MBT. Thus, evolution appears to have brought the same regulatory step under developmental control in very different organisms, but many factors complicate a comparison between systems, and here we emphasize that the detail of the dissection of the process in *Drosophila* has led us to a specific conclusion regarding Rif1 control of cell cycle slowing at the MBT in this organism.

In contrast to the early progressive slowing of the cell cycle in *Drosophila* embryos, the abrupt extension of cell cycle 14 shows features of a distinct regulatory transition. It has switch-like features [64]. The triggering of this sudden extension of S phase is not simply due to a limitation of a factor, because unlike the earlier progressive extension of S phase, inhibition of transcription prevents the abrupt extension [26]. This shows that existing materials are adequate to support another relatively fast cycle and that the extension of cycle 14 involves an active process. And importantly, we have previously shown that the key event in triggering the extension is the down-regulation of cyclin:Cdk1 [31][50]. Here, we show that it also depends on the positive action of Rif1 as a repressor of replication and that this activity of Rif1 depends on the down-regulation of Cdk1. In summary, we suggest that there are 2 distinct phases of S phase prolongation operating in the *Drosophila* embryo, one progressive and earlier and one abrupt and occurring at the onset of cycle 14. The *rif1* mutant is defective in the abrupt event that marks the MBT.

### Implications of the dispensability of Rif1

We found it surprising that *rif1* mutants have a highly penetrant phenotype with a specific failure to prolong S phase 14 yet produce viable progeny. This means that the prolongation of S phase 14 is not essential and that subsequent contributions of *rif1* to development and survival are also dispensable. We would like to put this in context.

The mutation of *rif1* was not without major consequences. Both hatching of maternally deficient embryos and survival of zygotically deficient flies were compromised. So *rif1* appears to be important, but a detailed characterization will be required to understand its contributions to survival. Nonetheless, it is clear that mutant embryos with a 27 min S phase 14 instead of the normal S phase duration of more than an hour can hatch. *rif1* mutant embryos still undergo an MBT, and the majority of the embryos slow their cell cycle because they arrest in a G2 after their abnormally fast S phase 14. A minority of the embryos undergoes a complete or partial extra syncytial cycle, suggesting that S phase timing contributes to regulation that normally introduces a G2 into the cell cycle at the MBT with great reliability.

The late-replication program in cell cycle 14 precedes obvious appearance of heterochromatic marks on satellite sequences [30]. These marks do appear later in cell cycle 14, and their appearance was not compromised in the absence of Rif1. Thus, neither the selective localization of Rif1 to satellite sequences nor the specific time of replication of the satellites in cycle 14 is required to trigger or guide the appearance of the heterochromatic marks. Importantly, we showed previously that recruitment of HP1a and associated acquisition of other heterochromatin marks delay replication of the X-chromosomal 359-satellite repeat in cycle 15 [30]. Here we show that in the absence of Rif1, the 359 repeat still exhibits late replication in cycle 15, suggesting that heterochromatin can act by a Rif1-independent pathway to cause replication delay. While *rif1* mutant embryos may show subtle alterations in the program of late replication in later cycles, presently the unique dependency of replication timing on Rif1 function appears limited to a narrow window of developmental time. Thus, survival of *rif1* mutants does not assess the importance of late replication per se, only the impact of the delay in cycle 14.

### Concluding remarks

Embryonic development presents early cell cycles with an unusual challenge—regulating cell division in the absence of transcription. Perhaps because of this early constraint, the biology of early embryos is streamlined and lacks many regulatory processes that appear later in development. When development introduces complications, it does so incrementally, highlighting individual regulatory circuits. By focusing on early development, we have been able to isolate and study the contributions of Rif1 to late replication and to S phase length. Our work uncovers a special developmental function for Rif1 in controlling the timely prolongation of S phase at the MBT and provides new insight into the control of late replication.

## Materials and methods

### Fly stocks

All *D*. *melanogaster* stocks were cultured on standard cornmeal-yeast medium. The following fly strains were used in this study: *w*^*1118*^ Canton-S (wild type), His2Av-mRFP (Bloomington stock number 23650 or 23651), mRFP-HP1a (30562), eyeless-gal4 (5535), maternal tubulin-gal4 (7063), UAS-shRNA^cdc7^ (TRiP GL00585), *mei41*^*D3*^ (gift from Tin Tin Su, University of Colorado Boulder), mCherry-PCNA (this study), rif1-EGFP (this study), cdc7-EGFP (this study), *rif1*^*-*^ (this study), UASp>Rif1-3xFlag (this study), UASp>Rif1-15a-3xFlag (this study).

### Generation of transgenic lines described in this study

All transgenesis injections were performed by Rainbow Transgenic Flies, Camarillo, CA.

The stocks rif1-EGFP and cdc7-EGFP were generated using CRISPR-Cas9 editing to modify the endogenous *rif1* and *cdc7* genes (diagrammed in S1 Fig) as described in [65]. Briefly, a single CRISPR target site was selected as close to the stop codon as possible. To direct homologous repair, approximately 1.5 kb of DNA on either side of the cut site was amplified from gDNA isolated from the vasa-Cas9 stock (51324). Both homology arms, a 6xGlyGlySer-EGFP tag, and the endogenous 3’ UTR were cloned into the pHD-DsRed vector using Gibson Assembly. To generate gRNA plasmids, annealed DNA oligos were cloned into the pU6-BbsI-chiRNA vector. The donor plasmid and the gRNA plasmid were coinjected into vasa-cas9 embryos by Rainbow Transgenic Flies. After screening for DsRed^+^ progeny, successful modification was confirmed by PCR genotyping and anti-GFP immunoblotting. A single male containing a successful modification was backcrossed to our lab’s *w1118* Canton-S stock for 5 generations to establish a stock.

CRISPR-Cas9 editing was used to generate the *rif1* null allele (S4 Fig) by replacing the *rif1* ORF with a visible 3xP3-DsRed marker. Two CRISPR target sites were selected, one site directly upstream of the start codon and one site 14 bp upstream of the stop codon. Gibson assembly was used to construct a homologous repair donor plasmid containing approximately 1.5 kb of DNA homologous to the genomic sequence directly upstream of the first cut site, the DsRed marker, and approximately 1.5 kb of DNA homologous to the genomic sequence directly downstream of the second cut site. Both gRNA plasmids and the donor plasmid were coinjected into vasa-cas9 (51324) embryos by Rainbow Transgenic Flies. After screening for DsRed^+^ progeny, successful replacement of the *rif1* ORF was confirmed by PCR genotyping (S4 Fig) and by Sanger sequencing across the recombinant breakpoints. A single male carrying the *rif1* null allele was backcrossed to our lab’s wild-type strain for 5 generations to establish the *rif1*^*-*^ stock.

To generate the mCherry-PCNA transgenic stock, approximately 1 kb of sequence upstream of the *pcna* start codon, the mCherry-5xGlyGlySer tag, and the pcna gene were cloned into the pw+attB vector using Gibson Assembly. The resulting transgenesis plasmid and phiC31 mRNA were coinjected into attP-9a embryos (9744) to perform phiC31-mediated integration.

To generate the UAS-Rif1.WT-3xFlag and UAS-Rif1.15a-3xFlag transgenic lines, the *rif1* ORF was amplified from embryo cDNA and inserted into the pENTR-D vector by TOPO cloning. In order to generate the phosphomutant Rif1.15a, a DNA fragment containing the mutated sequence was synthesized (Integrated DNA Technologies, Redwood City, CA) and used to replace the corresponding wild-type sequence in the ENTRY plasmid by Gibson Assembly. The ENTRY vectors were then recombined with the pPWF-attB construct using the LR Clonase II kit (Invitrogen, Carlsbad, CA). The resulting transgenesis plasmids and phiC31 mRNA were coinjected into attP-9a embryos (9744) to perform phiC31-mediated integration.

Plasmids and DNA sequences are available upon request.

### Embryo staining

Embryos were collected on grape agar plates, washed into mesh baskets, and dechorionated in 50% bleach for 2 min. Embryos were then devitellenized and fixed in a 1:1 mixture of methanol-heptane before storing in methanol at −20 °C. Embryos were gradually rehydrated in a series of increasing PTx:Methanol mixtures (1:3, 1:1, 3:1) before washing for 5 min in PTx (PBS with 0.1% Triton). Embryos were then blocked in PBTA (PTx supplemented with 1% BSA and 0.2% Azide) for 1 h at room temperature. Blocked embryos were then incubated with the primary antibody overnight at 4 °C. The following primary antibodies were used: rabbit anti-GFP at 1:500 (Invitrogen A11122) and mouse anti-tubulin at 1:100 (DSHB 12G10, AA12.1-s, and AA4.3-s). Embryos were then washed with PTx 3 times for 15 min each and then incubated with the appropriate fluorescently labeled secondary antibody (Molecular Probes) at 1:300 for 1 h in the dark at room temperature. Embryos were then washed again with PTx 3 times for 15 min each. In order to detect total DNA, DAPI was added to the second wash. Finally, stained embryos were mounted on glass slides in Fluoromount.

To detect both Rif1-EGFP and the AATAC satellite repeat, formaldehyde-fixed embryos were blocked in PBTA for 1 h, incubated with rabbit anti-GFP at 1:500 (Invitrogen A11122) for 1 h at room temperature, washed 3 times for 15 min each in PTX, incubated with fluorescently labeled secondary antibody at 1:300 for 1 h at room temperature in the dark, and then washed 3 times for 15 min in PTX. Embryos were then post-fixed in 4% formaldehyde for 20 min. FISH was then performed as previously described [26] using a Cy5 labeled AATAC 30-mer oligo probe (IDT).

### Protein extract preparation and immunoprecipitation

Protein extracts were prepared by homogenizing embryos in embryo lysis buffer (50 mM Tris-HCl pH 8.0, 100 mM NaCl, 1% Triton X-100, 1 mM EDTA) supplemented with 1X protease inhibitor cocktail (Pierce), PMSF, 100 mM Sodium Fluoride, 100 mM β-gylcerophosphate, and 10 mM sodium pyrophosphate. After lysis, 2X Sample Buffer was added to the extract, and samples were boiled for 5–10 min. For dephosphorylation reactions, protein extracts were prepared as above except for the omission of phosphatase inhibitors. Extracts were then supplemented with 1 mM MnCl_2_ and incubated with ƛ phosphatase for 30 min at 30 °C. The reaction was terminated by adding 2X Sample Buffer and boiling for 5–10 min.

For immunoprecipitation of GFP-tagged Rif1, dechorinated embryos were lysed using a dounce homogenizer in 500 μl of ice-cold RIPA buffer (50 mM Tris-HCl pH 8.0, 150 mM NaCl, 1% Triton X-100, 0.5% Sodium deoxycholate, 0.1% SDS, 1 mM DTT) supplemented with 1X protease inhibitor cocktail (Pierce, Waltham, MA), PMSF, 100 mM Sodium Fluoride, 100 mM β-gylcerophosphate, and 10 mM sodium pyrophosphate. The resulting extract was cleared by spinning twice at 12,000 rpm for 10 min each at 4 °C, after which the supernatant was incubated with 20 μl of GFP-TRAP Magnetic beads (Chromotek, Martinsried, Germany) for 1 h at 4 °C on a nutator. The beads were then washed 3 times with lysis buffer using a magnetic rack, and proteins were eluted by boiling in 30 μl of 2x Sample Buffer.

### Western blotting

For standard western blotting, protein extracts were separated by electrophoresis in precast 4%–15% polyacrylamide gels (Biorad, Hercules, CA). To separate phosphorylated forms of Rif1, protein extracts were run on a 0.5% agarose-strengthened 3% polyacrylamide gel containing 20 μM Mn^2+^-Phostag (AAL-107, Wako Chemicals, Japan). Proteins were then transferred to a PVDF membrane using a wet-transfer system. The membrane was blocked in TBST (TBS with 0.1% Tween-20) supplemented with 2.5% BSA and then incubated in the appropriate primary antibody at a dilution of 1:1,000 overnight at 4 °C. The blot was then washed 3 times for 10 min each in TBST and then incubated in the appropriate secondary antibody at a dilution of 1:10,000 for 1 h at room temperature. The blot was then washed 3 times for 10 min each in TBST and then treated with Pierce SuperSignal West Pico ECL and used to expose autoradiography film. The following antibodies were used: rabbit anti-GFP (ab290, Abcam, Cambridge, United Kingdom), rabbit anti-PP1a (2582, Cell Signaling, Danver, MA), and Goat anti-Rabbit HRP conjugated (Biorad).

### Microinjection

Embryo microinjections were performed as previously described [31]. In vitro transcribed mRNA was prepared using the CellScript T7 mRNA production system (CellScript, Madison, WI) as previously described [31] and injected at a concentration of 600 ng/μl. The purified proteins used in this study were described in [30].

### Microscopy

For live imaging, embryos were collected on grape agar plates and aged at 25 °C when appropriate. After dechorination in 50% bleach, embryos were aligned and glued to glass coverslips and then covered in halocarbon oil before imaging. Embryos were imaged using a spinning disk confocal microscope, and the data were analyzed using Volocity 6 (Perkin Elmer, San Jose, CA). For most experiments, approximately 30 embryos were watched under the microscope, and only the embryos at the appropriate developmental stage were filmed. When comparing different conditions, all images acquired in a single experiment were acquired at the same time and with identical microscope settings. Choice of objective and z-stack size were determined by experimental need. Unless otherwise noted, all images shown are projections of a z-stack series. When imaging embryos following microinjection, the objective was centered in the portion of the embryo nearest the site of injection to record the area of maximum effect.

### Reproducibility

The development of embryos is highly stereotyped, and the quality of the data is founded in the resolution of the imaging. Still images and movies presented in this paper are representative of multiple embryos filmed during a single experiment (technical replicates) and of identical experiments performed on embryos collected from independently sorted flies (biological replicates). Choice of embryos for imaging was dictated by developmental stage and by embryo health (unfertilized and clearly damaged embryos were excluded). Western blots reported in this paper were performed in 2 biological replicates (separate protein extracts from independent embryo collections).

## Supporting information

S1 Fig Generation and characterization of endogenously tagged Rif1::EGFP flies. (A) Schematic showing the gene structure of *drif1* and the CRISPR-Cas9 tagging strategy. Briefly, a single guide RNA was chosen to direct Cas9 cleavage in the extreme C-terminus of the *rif1* ORF. A donor plasmid containing approximately 1.5 kb of homology to either side of the break point was used to insert a GlyGlySer (linker)-EGFP tag in addition to a DsRed-selectable marker under the control of the eye-specific 3xP3 enhancer. (B) Anti-GFP western blot on embryonic protein extract to confirm successful tagging. (C) Still frames from time-lapse confocal imaging of embryos produced from the indicated crosses. Selected images are from individual embryos at 10 min into interphase of cycle 14. The Rif1 protein present at the MBT is maternally provided. Cas9, CRISPR-associated protein 9; CRISPR, clustered regularly interspaced short palindromic repeat; DsRed, *Discosoma* red fluorescent protein; EGFP, enhanced green fluorescent protein; MBT, mid-blastula transition; Rif1, Rap1 interacting factor 1.
(TIF)

S2 Fig (A) Injection of geminin eliminates S phase 14 foci of mCherry-PCNA. In control embryos, mCherry-PCNA marks nuclear locations of active DNA replication, resulting in bright PCNA foci later in S phase. When pre-RC formation is blocked by the injection of purified geminin protein during interphase 13, the nuclear PCNA signal is overall less intense and never resolves into replication foci. We conclude that transgenic mCherry-PCNA faithfully marks replicating sequences. (B) Stills from time-lapse imaging of Rif1-EGFP and mCherry-PCNA during the start of S phase 15. Note that the recruitment of Rif1 precedes the recruitment of PCNA to chromatin. Once S phase begins, PCNA is spread throughout the early replicating euchromatic portion of the nucleus, but the PCNA signal does not overlap with the Rif1-bound late-replicating heterochromatin, which by cycle 15 is concentrated to one edge of the nucleus. EGFP, enhanced green fluorescent protein; PCNA, proliferating cell nuclear antigen; pre-RC, pre-replicative complex; Rif1, Rap1 interacting factor.
(TIF)

S3 Fig Analysis of potential Rif1 CDK and DDK phosphorylation sites. (A) Multiple sequence alignment of the indicated portions of the Rif1 protein sequences from *Aedes aegypti* (1345–1541), *D*. *melanogaster* (946–1103), and *Musca domestica* (1041–1189) using Clustal Omega. Potential DDK and CDK phosphorylation sites are highlighted in red. Both CDK and DDK are serine/threonine kinases in which specificity is encoded by the residue in the +1 position. CDK phosphorylates S/T residues followed by a proline. DDK targets S/T residues followed by an acidic group, which can be provided by an acidic amino acid (D or E) or by a previous phosphorylation (e.g., in the sequence SSP). PP1 interaction motifs are highlighted in purple. (B) Analysis of the *D*. *melanogaster* Rif1 protein sequence using the PONDR tool to score for regions of intrinsic disorder. PONDR scores above 0.5 suggest regions of intrinsic disorder. Above the graph is a schematic of the relevant regions of the Rif1 protein. The N-terminal heat repeats are represented by the yellow box, and the portion of Rif1 containing the potential CDK and DDK phosphorylation sites analyzed in (A) is represented by the red box. CDK, cyclin-dependent kinase; DDK, Dbf4-dependent kinase; PONDR, Predictor of Natural Disordered Regions; PP1, protein phosphate 1; Rif1, Rap1 interacting factor 1.
(TIF)

S4 Fig (A) Schematic showing the gene structure of *Drosophila Rif1* and the CRISPR-Cas9 editing strategy used to generate the *rif1*-null allele. Briefly, 2 CRISPR target sites were selected, one site directly upstream of the start codon and one site directly upstream of the stop codon. Approximately 1.5 kb of DNA homologous to the genomic sequence either upstream or downstream of the break points was used to direct the replacement of the rif1 ORF with the visible 3xP3-DsRed marker. (B) Confirmation of correct replacement of the *rif1* ORF by PCR. Cas9, CRISPR-associated protein 9; CRISPR, clustered regularly interspaced short palindromic repeat; DsRed, *Discosoma* red fluorescent protein; Rif1, Rap1 interacting factor 1.
(TIF)

S1 Movie Rif1 forms dynamic nuclear foci as the embryonic cell cycle lengthens. This video accompanies Fig 1D. Live imaging of an Rif1-GFP (green) and H2aV-RFP (magenta) in an embryo completing the blastoderm cell cycles and the MBT. Video begins at mitosis 10 and ends in S phase 15 (post-MBT). In each cycle, green Rif1 foci appear upon exit from mitosis, disappear progressively as interphase progresses, and reform in the next cycle. With each cycle, Rif1 foci become more numerous and more persistent in parallel with the slowing of the cell cycle. Imaging is at the ventral midline, and at the onset of gastrulation (late during cycle 14), dramatic movements are associated with invagination of the ventral furrow. Toward the end of the movie, cells that flanked the invaginated furrow (domain 14 cells) divide and are evident as a row of paired cells running laterally along the ventral midline and featuring Rif1 foci. These cells are now in S phase 15 and are bordered on either side by cells still in G2 of the previous cycle. Z stacks were acquired every 1.5 min on a 100× oil objective. GFP, green fluorescent protein; His2Av, histone 2A variant; MTB, mid-blastula transition; RFP, red fluorescent protein; Rif1, Rap1 interacting factor.
(MOV)

S2 Movie Imaging the appearance and disappearance of Rif1 S phase foci. This video accompanies Figs 1E and 3. Live imaging of Rif1-GFP (green) and His2Av-RFP (magenta) at high frame rate (every 20 s) using a 100× oil objective during cycles 12, 13, and 14. Because of the obvious photobleaching using this imaging protocol, each cell cycle is a separate imaging experiment done on a different embryo. Low-level binding of Rif1 is evident on the chromosomes during anaphase, but obvious foci become apparent only after the beginning of interphase. When Rif1 dissociates from the chromatin, the focus of Rif1-GFP erodes from the outside over the course of 2 min as the signal fades. Note that for the cycle 14 sample, the movie ends in the middle of S phase 14. GFP, green fluorescent protein; His2Av, histone 2A variant; RFP, red fluorescent protein; Rif1, Rap1 interacting factor.
(MP4)

S3 Movie Coordination of Rif1 dissociation and DNA replication. This video showing Rif1-GFP (green) and mCherry-PCNA (purple) during S phase 14 accompanies Fig 2A. Movie starts at the end of Mitosis 13 and follows nuclei through S phase 14 and ends shortly after mitosis 14 in domain 5. Gastrulation movements are obvious at the 1-h mark, and the focus was manually adjusted at 01:08 to keep nuclei in frame. Rif1 dissociates from chromatin before the underlying sequences acquire PCNA signal, indicating onset of late replication. Absence of coincidence of the 2 signals is evident throughout the sequence. While complicated by nuclear movements, recruitment of PCNA to previously Rif1-staining regions is apparent near the close of S phase. Z stacks were acquired every 2 min on a 100× oil objective. GFP, green fluorescent protein; PCNA, proliferating cell nuclear antigen; Rif1, Rap1 interacting factor 1.
(MOV)

S4 Movie Rif1 is recruited to the late-replicating heterochromatin during mitosis 14 before the beginning of S phase 15. This video accompanies Fig 2B. Live imaging of Rif1-GFP (green) and mCherry-PCNA (magenta) during mitosis 14 and the start of S phase 15. Rif1 binds to the chromatin during mitotic exit and is enriched at the leading edge of the chromosome mass where the late-replicating pericentric heterochromatin resides. Rif1 binding is evident before the appearance of PCNA signal on the chromatin, which marks the start of S phase. Once S phase begins, the Rif1-stained heterochromatin does not overlap with the PCNA-stained early-replicating euchromatin. Z stacks were acquired every 20 s on a 100× oil objective. GFP, green fluorescent protein; PCNA, proliferating cell nuclear antigen; Rif1, Rap1 interacting factor.
(MOV)

S5 Movie Coordination of Rif1 dissociation and DNA replication during late S phase 15. This video shows Rif1-GFP (green) and mCherry-PCNA (magenta) during late S phase 15 as the heterochromatic chromocenter begins and completes DNA replication. Filming began after PCNA had invaded the apical portion of the nucleus containing the heterochromatin. Note that sequences marked by Rif1 are not also marked by PCNA. As S phase proceeds, Rif1 foci disappear and are replaced by PCNA as the underlying DNA replicates. Z stacks were acquired every 1 min on a 100× oil objective. GFP, green fluorescent protein; PCNA, proliferating cell nuclear antigen; Rif1, Rap1 interacting factor 1.
(MOV)

S6 Movie Imaging Rif1 and the replication of the 1.686 satellite repeat. This video accompanies Fig 2C. Live imaging of Rif1-GFP (green) and the late replicating satellite 1.686 using an mCherry labeled TALE-light protein (magenta) during S phase 13. Before 1.686 replicates, it is marked by Rif1 (coincident signal appears white). Rif1 then dissociates (at 00:04:50) before this repeat decondenses (at 00:05:30) and replicates. After replication, the TALE-light signal appears brighter and recompacted (00:09:50) but lacks an Rif1 signal. Finally, the 1.686 loci separate, align, and then lose fluorescence upon progression to mitosis. Z stacks were acquired every 20 s on a 100× oil objective. GFP, green fluorescent protein; Rif1, Rap1 interacting factor 1; TALE, transcription activator-like effector.
(MOV)

S7 Movie Ectopic activation of Cdk1 during S phase 14 drives Rif1 dissociation and accelerates late replication. This video accompanies Fig 4B. Live imaging of Rif1-GFP (green) and mCherry-PCNA (magenta) during S phase 14 following injection of Cdc25 mRNA during cycle 13. Introduction of this Cdk1 activator accelerates the loss of Rif1 foci and drives the underlying sequences to replicate earlier in S phase (S3 Movie). Z stacks were acquired every 2 min on a 100× oil objective. Cdc7, cell division cycle 7; Cdk1, cyclin-dependent kinase 1; GFP, green fluorescent protein; PCNA, proliferating cell nuclear antigen; Rif1, Rap1 interacting factor.
(MOV)

S8 Movie *rif1* embryos undergo an early mitosis 14. This video accompanies Fig 6C. Live imaging of GFP-HP1a injected into in a wild-type embryo (left) and a *rif1* embryo (right). Movies begin in cycle 12 and follow embryos through cycle 14. Note that the *rif1* embryo undergoes an extra synchronous mitosis approximately 23 min into interphase 14. Z stacks were acquired at intervals of 1 min and 20 s on a 20× air objective. GFP, green fluorescent protein; HP1a, heterochromatin protein 1a; *rif1*, Rap1 interacting factor 1.
(MP4)

S9 Movie Level of nuclear Cdc7 decline over the blastoderm cycles leading up to the MBT. This video accompanies Fig 7B. Live imaging of Cdc7-GFP (green) and His2Av-RFP (magenta) during cell cycles 11 through 14. Z stacks were acquired every minute on a 20× air objective. Cdc7, cell division cycle 7; GFP, green fluorescent protein; His2Av, histone 2A variant; MBT, mid-blastula transition; RFP, red fluorescent protein.
(MOV)

S1 Data Underlying data for Figs 4c and 6A. (XLSX)

## Abbreviations

ATR: ataxia telangiectasia and Rad3-related protein
Cas9: clustered regularly interspaced short palindromic repeat–associated protein 9
Cdc25: cell division cycle 25
Cdc7: cell division cycle 7
CDK: cyclin-dependent kinase
Cdk1: cyclin-dependent kinase 1
CRISPR: clustered regularly interspaced short palindromic repeat
DDK: Dbf4-dependent kinase
Drf1: Dbf4 related factor 1
dsDNA: double-stranded DNA
EGFP: enhanced green fluorescent protein
FISH: fluorescence in situ hybridization
GFP: green fluorescent protein
H3K9me: histone H3 methylated on lysine 9
His2Av: histone 2A variant
HP1: heterochromatin protein 1
IP: immunoprecipitation
MBT: mid-blastula transition
MCM: minichromosome maintenance protein
MTD: maternal triple driver
ORC: origin recognition complex
Orc2: origin recognition complex 2
ORF: open reading frame
PCNA: proliferating cell nuclear antigen
PP1: protein phosphatase 1
pre-RC: pre-replicative complex
RFP: red fluorescent protein
Rif1: Rap1 interacting factor 1
RNAi: RNA interference
TALE: transcription activator-like effector
UAS: upstream activating sequence
