# Supplementary figures and images for: Rif1 prolongs the embryonic S phase at the Drosophila mid-blastula transition
Source: PLoS Biol. 2018 May 10;16(5):e2005687. doi: 10.1371/journal.pbio.2005687 (PMC5963817; doi:10.1371/journal.pbio.2005687)

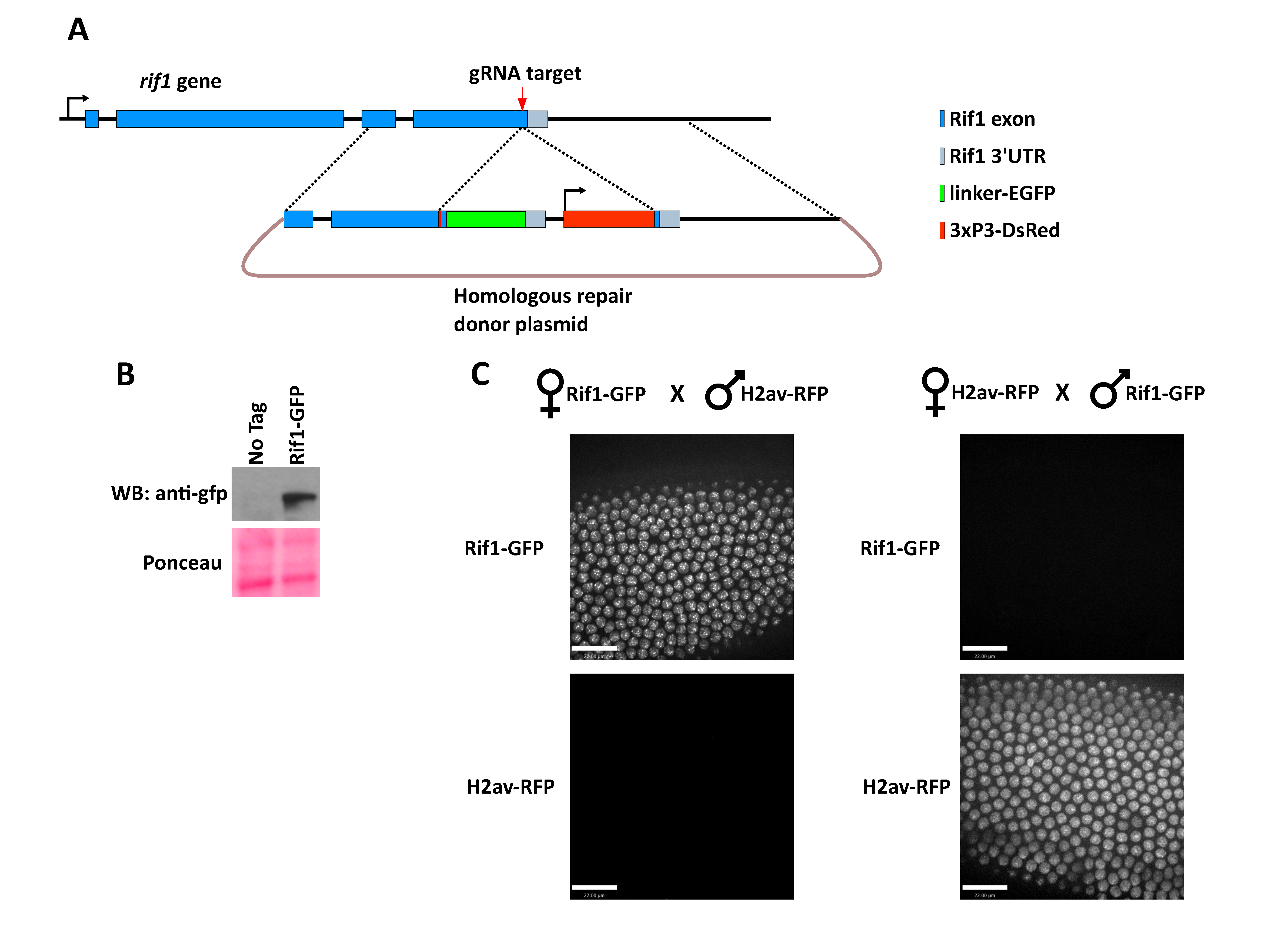

Supplement: S1 Fig — (A) Schematic showing the gene structure of drif1 and the CRISPR-Cas9 tagging strategy. Briefly, a single guide RNA was chosen to direct Cas9 cleavage in the extreme C-terminus of the rif1 ORF. A donor plasmid containing approximately 1.5 kb of homology to either side of the break point was used to insert a GlyGlySer (linker)-EGFP tag in addition to a DsRed-selectable marker under the control of the eye-specific 3xP3 enhancer. (B) Anti-GFP western blot on embryonic protein extract to confirm successful tagging. (C) Still frames from time-lapse confocal imaging of embryos produced from the indicated crosses. Selected images are from individual embryos at 10 min into interphase of cycle 14. The Rif1 protein present at the MBT is maternally provided. Cas9, CRISPR-associated protein 9; CRISPR, clustered regularly interspaced short palindromic repeat; DsRed, Discosoma red fluorescent protein; EGFP, enhanced green fluorescent protein; MBT, mid-blastula transition; Rif1, Rap1 interacting factor 1. (TIF) [file pbio.2005687.s001.tif]

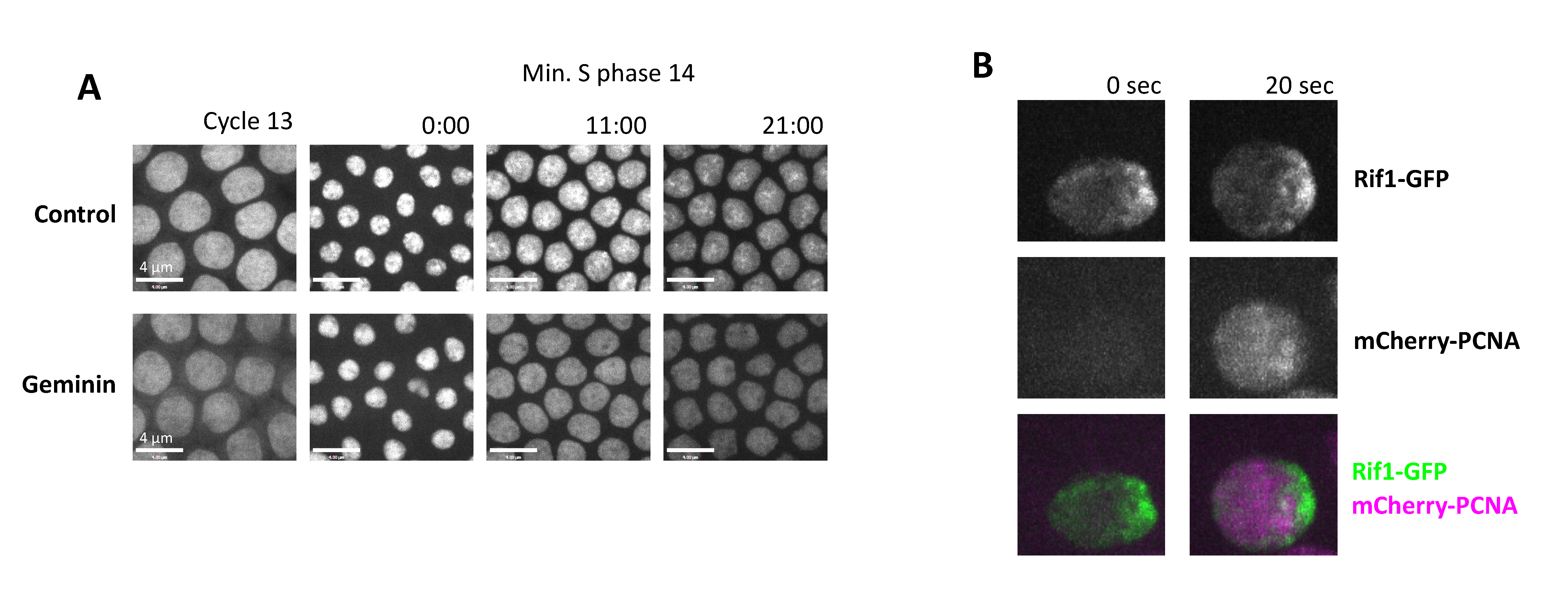

Supplement: S2 Fig — (A) Injection of geminin eliminates S phase 14 foci of mCherry-PCNA. In control embryos, mCherry-PCNA marks nuclear locations of active DNA replication, resulting in bright PCNA foci later in S phase. When pre-RC formation is blocked by the injection of purified geminin protein during interphase 13, the nuclear PCNA signal is overall less intense and never resolves into replication foci. We conclude that transgenic mCherry-PCNA faithfully marks replicating sequences. (B) Stills from time-lapse imaging of Rif1-EGFP and mCherry-PCNA during the start of S phase 15. Note that the recruitment of Rif1 precedes the recruitment of PCNA to chromatin. Once S phase begins, PCNA is spread throughout the early replicating euchromatic portion of the nucleus, but the PCNA signal does not overlap with the Rif1-bound late-replicating heterochromatin, which by cycle 15 is concentrated to one edge of the nucleus. EGFP, enhanced green fluorescent protein; PCNA, proliferating cell nuclear antigen; pre-RC, pre-replicative complex; Rif1, Rap1 interacting factor. (TIF) [file pbio.2005687.s002.tif]

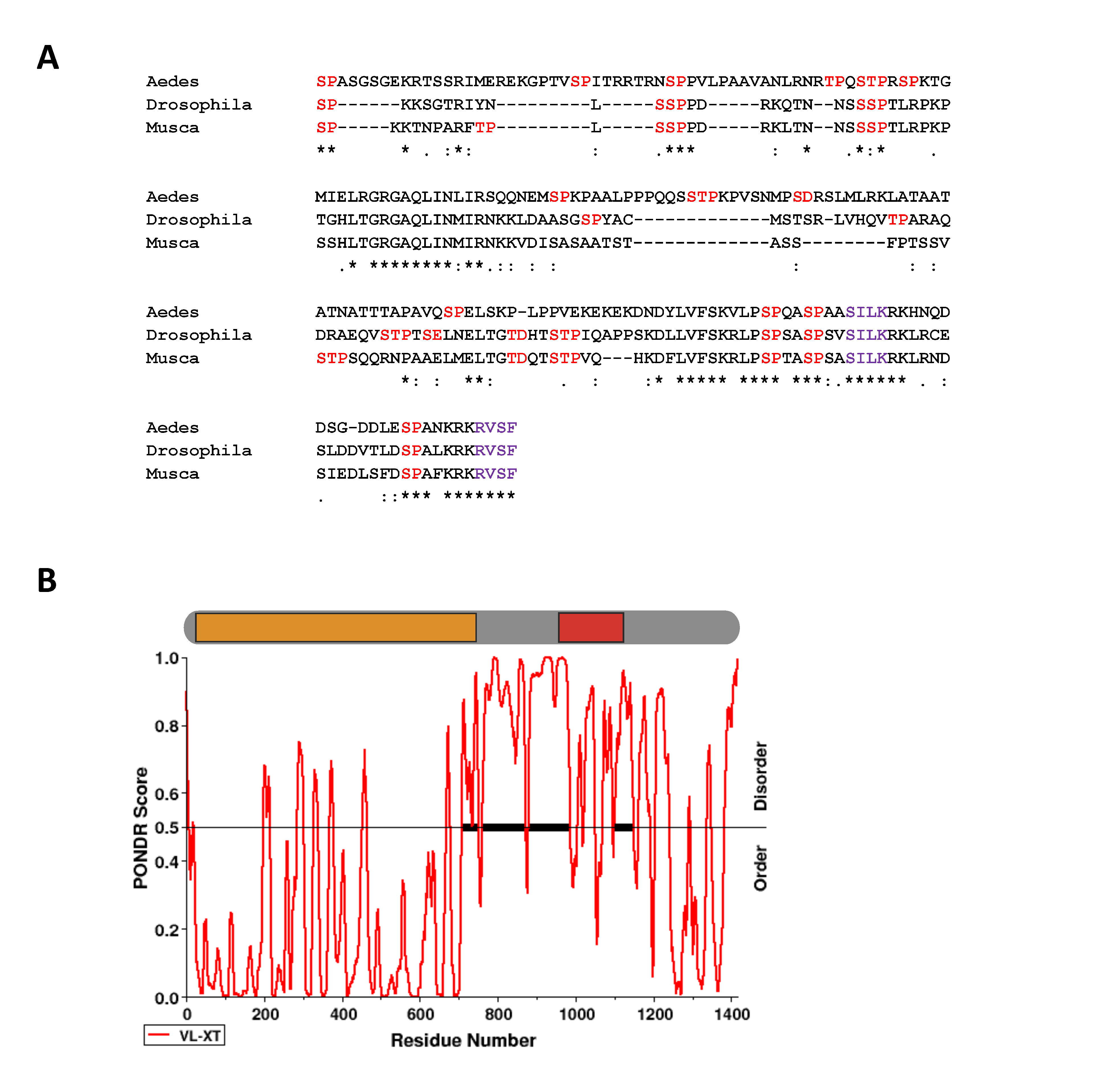

Supplement: S3 Fig — (A) Multiple sequence alignment of the indicated portions of the Rif1 protein sequences from Aedes aegypti (1345–1541), D. melanogaster (946–1103), and Musca domestica (1041–1189) using Clustal Omega. Potential DDK and CDK phosphorylation sites are highlighted in red. Both CDK and DDK are serine/threonine kinases in which specificity is encoded by the residue in the +1 position. CDK phosphorylates S/T residues followed by a proline. DDK targets S/T residues followed by an acidic group, which can be provided by an acidic amino acid (D or E) or by a previous phosphorylation (e.g., in the sequence SSP). PP1 interaction motifs are highlighted in purple. (B) Analysis of the D. melanogaster Rif1 protein sequence using the PONDR tool to score for regions of intrinsic disorder. PONDR scores above 0.5 suggest regions of intrinsic disorder. Above the graph is a schematic of the relevant regions of the Rif1 protein. The N-terminal heat repeats are represented by the yellow box, and the portion of Rif1 containing the potential CDK and DDK phosphorylation sites analyzed in (A) is represented by the red box. CDK, cyclin-dependent kinase; DDK, Dbf4-dependent kinase; PONDR, Predictor of Natural Disordered Regions; PP1, protein phosphate 1; Rif1, Rap1 interacting factor 1. (TIF) [file pbio.2005687.s003.tif]

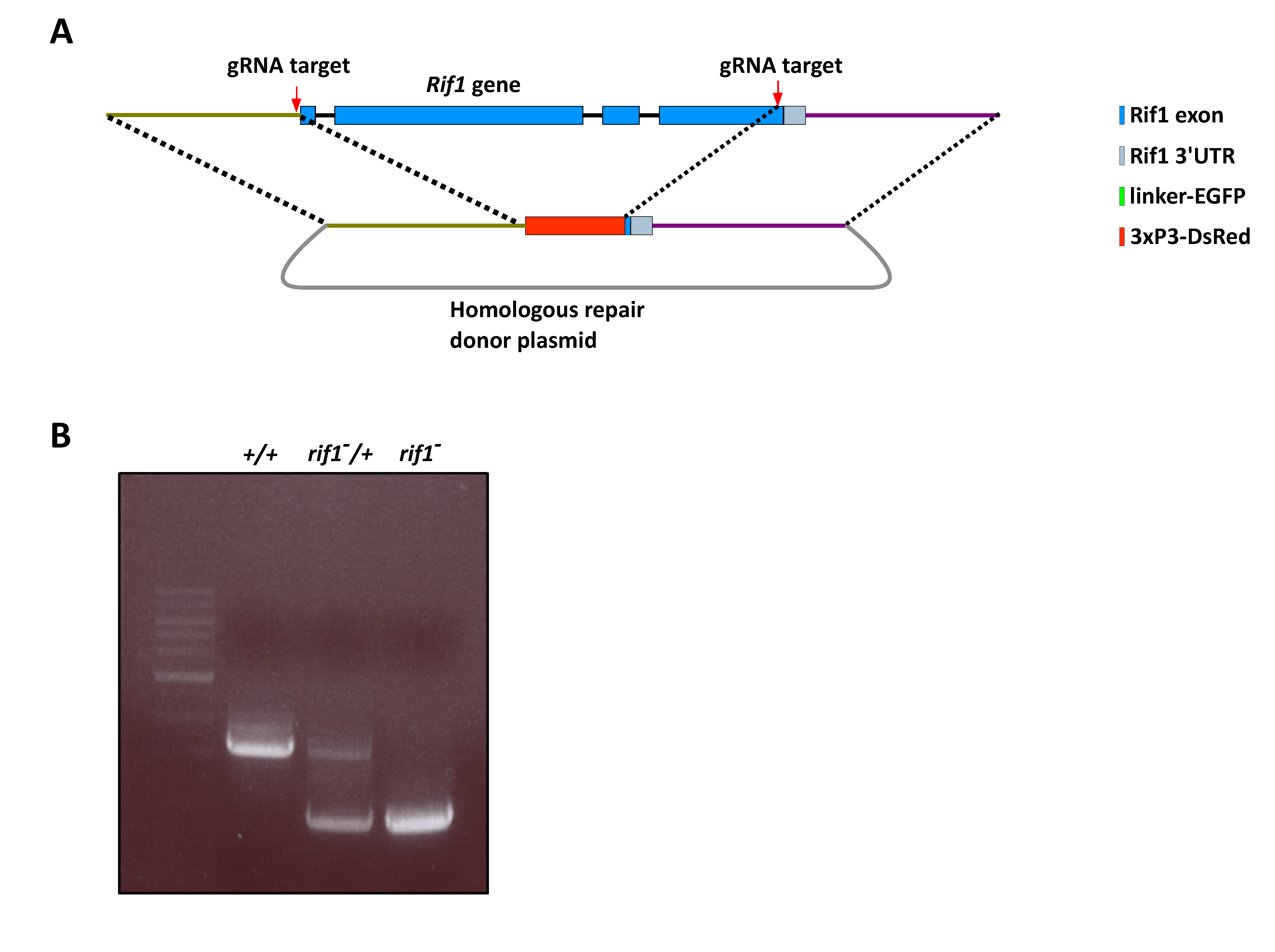

Supplement: S4 Fig — (A) Schematic showing the gene structure of Drosophila Rif1 and the CRISPR-Cas9 editing strategy used to generate the rif1-null allele. Briefly, 2 CRISPR target sites were selected, one site directly upstream of the start codon and one site directly upstream of the stop codon. Approximately 1.5 kb of DNA homologous to the genomic sequence either upstream or downstream of the break points was used to direct the replacement of the rif1 ORF with the visible 3xP3-DsRed marker. (B) Confirmation of correct replacement of the rif1 ORF by PCR. Cas9, CRISPR-associated protein 9; CRISPR, clustered regularly interspaced short palindromic repeat; DsRed, Discosoma red fluorescent protein; Rif1, Rap1 interacting factor 1. (TIF) [file pbio.2005687.s004.tif]
